# Ultrasonic washing as an abiotic elicitor to induce the accumulation of phenolics of fresh-cut red cabbages: Effects on storage quality and microbial safety

**DOI:** 10.3389/fnut.2022.1006440

**Published:** 2022-11-03

**Authors:** Chen Hong, Hong-Chang Zhou, Yi-Ming Zhao, Haile Ma

**Affiliations:** ^1^School of Food and Biological Engineering, Jiangsu University, Zhenjiang, China; ^2^Institute of Food Physical Processing, Jiangsu University, Zhenjiang, China

**Keywords:** ultrasonic washing, fresh-cut red cabbages, phenolic content, phenolic accumulation, storage quality, microbial safety

## Abstract

Ultrasonic washing has been proved to be an abiotic elicitor to induce the accumulation of phenolics in some fruit and vegetables. However, the feasibility of ultrasonic washing on the accumulation of phenolics in fresh-cut red cabbages has not yet been reported. Therefore, the effects of ultrasonic washing on the phenolics and related phenolic metabolism enzymes of fresh-cut red cabbages, as well as quality and microbial safety during cold storage, were investigated. Firstly, the single-factor tests were used to optimize the ultrasonic processing parameters, including frequency mode, frequency amplitude, power density, frequency cycle time, and ultrasonic washing. Then the activities of the enzymes related to phenolic metabolisms after optimal ultrasound treatment were investigated, including phenylalanine ammonia-lyase (PAL), polyphenol oxidase (PPO), and peroxidase (POD). Additionally, the quality and microbial safety of fresh-cut red cabbages stored at 4°C under the optimal ultrasound treatment were evaluated. The results showed that the content of soluble phenolics (SPs) in fresh-cut red cabbages increased significantly during storage under the optimal conditions (28 ± 2 kHz, 60 W/L, 400 ms, and 20 min) compared with the control (*P* < 0.05). The PAL activity was activated and the PPO and POD activities were inhibited after ultrasonic washing, which contributed to the increase in the content of SPs. Meanwhile, the storage quality and microbial safety of fresh-cut red cabbages were improved. Ultrasonic washing reduced the weight loss and respiration rate and improved the color and texture characteristics. Additionally, the fresh-cut red cabbages after ultrasonic washing showed more retention of ascorbic acid (AA), total soluble proteins (TSPs), total soluble sugars (TSSs), and total soluble solids (SSs) compared with the control. Finally, ultrasonic washing effectively inhibited the growth of bacteria, molds and yeasts, which is beneficial to the extension of the shelf-life of fresh-cut red cabbages. Therefore, ultrasonic washing can be used as a tool to increase the content of SPs in fresh-cut red cabbages while retaining quality attributes and microbial safety.

## Introduction

Red cabbages (*Brassica oleracea var. capitata f. rubra*), which belong to the Cruciferous family, are rich in phenolics (1, 2). As the largest category of phytochemicals in plant-based foods, phenolics are highly associated with the antioxidant ability of fruit and vegetables (3, 4). They can protect the human body against damage from reactive oxygen species, reducing the risk of chronic diseases such as cancer and cardiovascular diseases (5). Thus, red cabbages are good sources of natural antioxidants. Studies have shown that cooking methods such as boiling, steaming, stir-frying, and microwave processing can reduce the total phenolic content of red cabbages (6). In order to minimize the losses of phenolics, red cabbages are often made into fresh-cut vegetables and eaten as salads (7).

Washing is an essential step to ensure food safety in the process of fresh-cut fruit and vegetables. This process can remove impurities, including residual soils, debris, insects, pesticides, and microorganisms. Meanwhile, it can clean the juice released from the shredded fruit and vegetables to decrease the food corruption rate, improve edible quality, and prolong the shelf-life (8, 9). Currently, chemical washing methods are commonly used in the fruit and vegetable industry, especially disinfectants based on chlorine and chlorinated compounds due to their convenience, low cost, and high antimicrobial activity (9, 10). Nevertheless, chlorine-based compounds are corrosive, damaging human health by causing skin and respiratory tract irritation. Moreover, water hyperchlorination caused by these chemicals can produce carcinogens that are harmful to the environment, such as high-concentration trihalomethane (11). Therefore, emerging technologies, such as high pressure, electrolyzed water, pulsed electric field, irradiation, ozone, and ultrasound, have been considered alternatives in the food industry in recent years (12–15).

As a residual-free, safe, and eco-friendly novel technology, ultrasonic washing has been widely used in the postharvest preservation and storage of fruit and vegetables, such as strawberries, lettuces, red bell peppers, and cherry tomatoes (16, 17). It is well known that cavitation effects are the unique physical phenomena caused by the propagation of ultrasonic waves in liquids, including physical effects (e.g., shock waves, micro-jets, shear force, and turbulence) and chemical effects (i.e., the production of a large number of free radicals) (18). Evidences have shown that the cavitation effects can enhance the elution of soil, elimination of microorganisms, and degradation of pesticide residues when ultrasonic washing is applied to fruit and vegetables (19–21). Additionally, it has been also reported that the quality of fruit and vegetables can be effectively maintained by the change in the activities of endogenous enzymes when subjected to ultrasonic washing (11). More interestingly, bioactive compounds in fruit and vegetables can be remarkably affected during the washing process, such as phenolics, carotenoids, and ascorbic acid (AA) (13, 21), especially phenolics. Ultrasound has been considered an abiotic elicitor that induces the accumulation of phenolics in fruit and vegetables, such as broccoli florets (22), mangoes (23), and kiwifruit (24), which is conducive to the obtainment of functional fruit and vegetables with increased levels and bioavailability of nutraceuticals (25, 26). The accumulation of phenolics may be related to the regulation of phenolic metabolism enzymes after ultrasonic washing, such as phenylalanine ammonia-lyase (PAL), polyphenol oxidase (PPO), and peroxidase (POD) (22, 27). However, not all fruit and vegetables are applicable to ultrasonic washing such as fresh-cut tomatoes and carrots, which showed lower phenolic content than water washing (28, 29). Currently, there are few studies on ultrasonic washing applied to red cabbages. Therefore, the feasibility of ultrasound as an abiotic elicitor to induce the accumulation of phenolics in fresh-cut red cabbages remains to be investigated.

In this study, the effects of ultrasonic frequency mode, frequency amplitude, ultrasonic power density, frequency cycle time, and ultrasonic time on the phenolics in fresh-cut red cabbages were studied by single-factor tests. The activities of phenolic metabolism enzymes after ultrasound treatment were explored, including PAL, PPO, and POD. Meanwhile, the effects of ultrasonic washing on other quality parameters [weight, respiration rate, color, firmness, AA, total soluble proteins (TSPs), total soluble sugars (TSSs), and total soluble solids (SSs)] and microbial safety (total number of bacteria, molds and yeasts) during 4°C storage were investigated under optimized conditions.

## Materials and methods

### Raw materials

Fresh red cabbages (*Brassica oleracea var. capitata f. rubra*) at the maturity stage (100–120 days after sowing, i.e., the red cabbages were sowed in February or March and harvested in June or July) were obtained from local markets in Zhenjiang (China) and pre-selected based on size, color, and visual quality to ensure initial consistency. The red cabbages were placed in a refrigerator at 4°C until the experiment. Before processing, the three outer leaves and any other leaves with visible damage were discarded. Then the red cabbages were manually cut into pieces of approximately 0.5 cm × 5 cm by a sterile knife. The fresh-cut red cabbages were used in the following sections.

### Ultrasonic washing procedure

**FIGURE 1:**
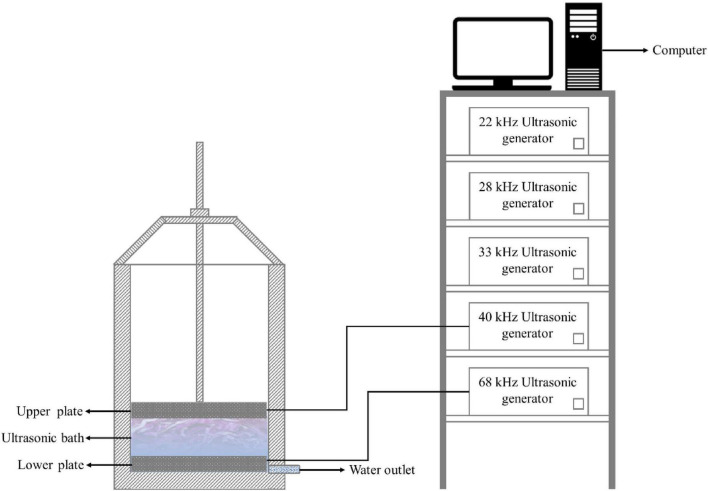
Schematic diagram of the flat-plate ultrasound equipment.

The fresh-cut red cabbages were washed with the flat-plate ultrasound equipment manufactured by Jiangsu University (as shown in Figure 1). The equipment can be operated by four frequency modes: single-fixed frequency, single-sweep frequency, double-fixed frequency, and double-sweep frequency. Single-frequency ultrasound is controlled by either the upper flat or lower flat. For dual-frequency ultrasound, both flats work at the same time. The maximum power of a single plate is 600 W. The sweep frequency model (α ± δ kHz) refers to the sweep frequency cycle of the increasing period from α − δ to α + δ kHz and the decreasing period from α + δ to α − δ kHz with the same linear speed in the form of an isosceles triangle. α is the central frequency of 22, 28, 33, 40, and 68 kHz. δ is the frequency amplitude, i.e., ±1 and ±2 kHz. An increasing period plus a decreasing period is defined as the cycle time of the sweep frequency. When the δ is set to zero, the operation is a fixed frequency model. The effects of ultrasound operating parameters on the phenolics of fresh-cut red cabbages were investigated by single-factor tests. The details, including ultrasonic frequency mode, frequency amplitude, ultrasonic power density, frequency cycle time, and ultrasonic time, are shown in Table 1. The operating parameters were selected based on the published reviews (14, 30) and previous studies in our research group (31, 32).

**TABLE 1:** Levels of ultrasonic frequency mode, ultrasonic power density, frequency amplitude, frequency cycle time, and ultrasonic time in the single-factor tests.

Factors	Levels
Ultrasonic frequency mode	Single-fixed frequency: 22, 28, 33, 40, and 68 kHz Single-sweep frequency: 22 ± 2, 28 ± 2, 33 ± 2, 40 ± 2, and 68 ± 2 kHz Dual-fixed frequency: 22/28, 22/33, 22/40, 22/68, 28/33, 28/40, 28/68, 33/40, 33/68, and 40/68 kHz Dual-sweep frequency: (22 ± 2)/(28 ± 2), (22 ± 2)/(33 ± 2), (22 ± 2)/(40 ± 2), (22 ± 2)/(68 ± 2), (28 ± 2)/(33 ± 2), (28 ± 2)/(40 ± 2), (28 ± 2)/(68 ± 2), (33 ± 2)/(40 ± 2), (33 ± 2)/(68 ± 2), and (40 ± 2)/(68 ± 2) kHz
Frequency amplitude	±1 and ±2 kHz
Ultrasonic power density	20, 40, 60, 80, and 100 W/L
Frequency cycle time	100, 200, 300, 400, and 500 ms
Ultrasonic time	10, 15, 20, 25, and 30 min

The same batch of red cabbages was used for the evaluation of the same factor with different levels to ensure the comparability of phenolic content, except for the factor of ultrasonic frequency mode. As there were 30 levels of frequency modes in the study, it was difficult to treat the same batch of fresh-cut red cabbages at one time. Therefore, different batches of red cabbages were used to study the effects of single-frequency and dual-frequency modes.

Fresh-cut red cabbages (500 g) were placed in an ultrasonic bath (36 cm × 29 cm × 100 cm) containing sterile distilled water for ultrasonic washing. The water temperature was kept at 20°C to avoid thermal effects. After treatment, excess water was removed with sterile filter paper from the surface of the samples and air-dried on a clean bench (OptiClean 1300, Heal Force Bio-Meditech Co., Shanghai, China). Then samples were placed in polystyrene containers and refrigerated at 4°C. They were taken every 2 days (0, 2, 4, 6, and 8 days) during storage for experimental analysis. Each experiment was repeated in triplicates. The results were expressed as means of three triplicates ± standard deviations. Fresh-cut red cabbages washed with sterile distilled water were used as the control.

### Determination of phenolic content

#### Extraction of soluble phenolics

The soluble phenolics (SPs) of the fresh-cut red cabbages were extracted by the method of Abdel-Aal et al. (33) with some modifications. Briefly, samples (30 g) were mixed with 300 mL of 80% chilled methanol. Then the mixtures were homogenized using a JYL-350S homogenizer (Joyoung Co., Ltd., Zhejiang, China) for 2 min. The supernatants were obtained by centrifugation at 10,000 rpm for 10 min for further analysis of SPs. Meanwhile, the sediments were used for the extraction of insoluble-bound phenolics (IPs).

#### Extraction of insoluble-bound phenolics

The IPs were extracted according to the method of Viacava et al. (34) with some modifications. Briefly, the sediments from the above extraction of SPs were hydrolyzed with 20 mL of 4 M NaOH at room temperature for 1 h with continuous shaking under nitrogen gas by a ZQZY-78CN shaker (Zhichu Biotechnology Co., Ltd., Shanghai, China). Then samples were acidified to pH 2 with 6 M HCl and centrifuged at 10,000 rpm for 10 min to obtain the supernatants. The supernatants were extracted with 20 mL ethyl acetate for 10 min, then centrifuged, and eventually dried under a nitrogen stream until dryness. The extracted sediments were redissolved in 5 ml of 80% methanol for the analysis of IPs.

#### Determination of the contents of soluble phenolics and insoluble-bound phenolics

The contents of SPs and IPs were quantified by the Folin-Ciocalteu method. The admixtures of 1 mL sample solution, 0.5 mL Folin-Ciocalteu reagent, 2 mL of 7.5% Na_2_CO_3_, and 6 mL distilled water were incubated at 70°C for 30 min. Then the absorbance of the solution was measured at 750 nm using a T6 UV/VIS spectrophotometer (Purkinje General Instrument Co., Ltd., Shanghai, China). The phenolic content was expressed as mg GAE/100 g FW fresh weight (note: GAE-gallic acid equivalents; FW-fresh weight).

Due to the differences in the initial contents of SPs in different batches of fresh-cut red cabbages used for frequency modes, it is difficult to directly compare the frequency modes by the content of SPs. Therefore, the phenolic retention rate under different frequency modes were used for the comparison. The retention rate of SPs was calculated as the following equation:


(1) Phenolic⁢retention⁢rate=Content⁢of⁢SPs⁢after⁢storageContent⁢of⁢SPs⁢in⁢the⁢control⁢group⁢on⁢Day⁢ 0

### Determination of the activities of phenylalanine ammonia-lyase, polyphenol oxidase, and peroxidase

For PAL activity measurement, fresh-cut red cabbages (30 g) were homogenized with 150 mL of chilled 0.05 M phosphate buffer solution (pH 7.8) for 2 min. Then the mixtures were centrifuged at 10,000 rpm for 10 min at 4°C, and the supernatant was collected to measure the PAL activity with an ELISA kit (Tongwei Reagent Co., Ltd., Shanghai, China) according to the manufacturer’s instructions. One unit of PAL activity was defined as the amount of enzyme that produced 1 μg cinnamic acid per hour.

For PPO and POD activity measurement, fresh-cut red cabbages (30 g) were homogenized with 150 mL of chilled 0.05 M phosphate buffer solution (pH 7.8) containing 1% polyvinylpyrrolidone for 2 min, and then centrifuged at 10,000 rpm for 10 min at 4°C. The supernatant was used for the following analysis of PPO and POD activities. The activities of PPO and POD were measured according to the method of Yeoh and Ali (27) with slight modifications. The assay of PPO activity was conducted by mixing 145 μL of 0.1 M catechol and 5 μL of supernatant. Absorbance was taken every 15 s for 4 min at 420 nm using an automatic microplate reader (Infinite 200 PRO, Tecan Trading AG, Switzerland). One unit of PPO activity was defined as the amount of enzyme that resulted in an increase of 0.01 absorbance unit per second. POD activity was measured by adding 1 μL of supernatant and 145 μL of 0.1 M sodium phosphate buffer (pH 6.0) blended with 20 mM guaiacol and 4 mM H_2_O_2_. The absorbance of the mixtures was recorded at 470 nm every 15 s for 4 min using the same automatic microplate reader as mentioned before. One unit of POD activity was defined as the amount of enzyme that resulted in an increase of 0.01 absorbance unit per second.

### Determination of weight retention rate and respiration rate

The weight retention rate of the samples was calculated as follows:


(2) Weight⁢retention⁢rate=Weight⁢after⁢storageWeight⁢in⁢the⁢control⁢group⁢on⁢Day⁢ 0

The respiration rate was measured by the F-900 Portable Analyzer (Felix instruments Co., Ltd., WA, United States). For each group, fresh-cut red cabbages (90 g) were placed in a 2-L sealed vessel in a well-ventilated environment at 25°C and kept sealed for 1 h. The respiration rate was measured by the changes in the concentration of CO_2_ and expressed as mg CO_2_⋅kg^–1^⋅h^–1^ (35).

### Determination of browning degree and cut surface color

The determination of the browning degree was based on the method of Li et al. (36) with some modifications. Fresh-cut samples (30 g) were homogenized with 300 mL of 80% ethanol. Then the mixtures were centrifuged at 10,000 rpm for 10 min to obtain the supernatant. The browning degree was expressed as the absorbance of the supernatant measured at 420 nm by a T6 UV/VIS spectrophotometer.

The color of the cut surface was measured by a CR-400 colorimeter (Konica Minolta, Japan) calibrated with a standard white plate. The CIELAB color coordinates L* (lightness), a* (green to red), and b* (blue to yellow) were collected at ten different spots from the cut surface, and the average values were reported. The total color difference (Δ*E*) was calculated according to the following equation:


(3) $$\Delta \,E=\sqrt{{\left(L^{*}-L_{0}^{*}\right)}^{2}+{\left(a^{*}-a_{0}^{*}\right)}^{2}+{\left(b^{*}-b_{0}^{*}\right)}^{2}}$$

Where $L_{0}^{*}$, $a_{0}^{*}$, and $b_{0}^{*}$ are the values of the standard white plate.

### Determination of firmness

Firmness was determined by an XT2i Texture analyzer (Stable Micro Systems, Godalming, United Kingdom) equipped with a P/2N probe with a diameter of 2 mm. Parameters referred to the method of Xu et al. (37) with some modifications and were set as follows: pretest speed, 1 mm/s; test speed, 1 mm/s; post-test speed, 10 mm/s; auto-trigger force, 5 g; and travel distance of the probe, 9 mm. The firmness was recorded with the maximum force (g) during penetration.

### Determination of the contents of ascorbic acid, total soluble proteins, total soluble sugars, and total soluble solids

Fresh-cut samples (30 g) were homogenized with 300 mL of 0.05 M oxalic acid containing 0.0002 M ethylene diamine tetraacetic acid, 0.05 M phosphate buffer solution (pH 7.8), 80% ethanol, and distilled water, respectively. The supernatant after centrifugation at 10,000 rpm for 10 min was used to measure the contents of AA, TSPs, TSSs, and SSs. The AA content was evaluated using phosphomolybdate-blue spectrophotometry (38). The contents of TSPs and TSSs were measured with the Bradford method and anthrone method using bovine serum albumin and glucose as a standard, respectively. The content of SSs was determined using a hand-held refractometer (ATAGO Tokyo, Japan).

### Microbiological analysis

The total number of bacteria, molds and yeasts in fresh-cut red cabbages were measured according to the method of Yildiz et al. (39) with some modifications. Samples (30 g) were immersed in a sterile saline solution of 300 mL, then homogenized using an LC-PJ-400M sterile homogenizer (Lichen-Bx Instrument Technology Co., Ltd., China) for 2 min. The homogenate was decimally diluted using sterile saline. Then the dilution solution (0.1 mL) was taken and surface plated onto plate count agar (Sinopharm Chemical Reagent Co., Ltd., Shanghai, China) at 37°C for 48 h and Bangladeshi-red culture medium (Sinopharm Chemical Reagent Co., Ltd., Shanghai, China) at 28°C for 96 h to count the total number of bacteria, molds and yeasts, respectively.

### Scanning electron microscopy of fresh-cut red cabbage leaves

In order to observe the anatomical characteristics (i.e., cross section and abaxial epidermis) of fresh-cut red cabbage leaves by a field emission scanning electron microscopy (Model Regulus8100, Hitachi, Japan), leaf samples were prepared according to the method of Marasek-Ciolakowska et al. (40) with some modifications. Fresh sample sections were fixed in 2.5% glutaraldehyde and then dehydrated in an ethanol series. Then the tissues were desiccated with critical point drying CO_2_ and sputter-coated with gold for observation.

### Statistical analysis

SPSS 19 (SPSS Inc., Chicago, IL, United States) and excel 2016 software were applied to perform the analysis of variance (ANOVA). Significant difference was determined among the treatments using Duncan’s test with a 95% level of confidence (*P* < 0.05). Origin 2018 and excel 2016 software were used in the graphical report.

## Results and discussion

### Analysis of ultrasound operating parameters

#### Effects of ultrasonic frequency mode on the contents of soluble phenolics and insoluble-bound phenolics

The effects of single-fixed and single-sweep frequency on the contents of SPs and IPs are shown in Supplementary Figure 1, and the effects of dual-fixed and dual-sweep frequency on the contents of SPs and IPs are displayed in Supplementary Figure 2. As can be seen from Supplementary Figures 1, 2, the content of SPs in the red cabbage extracts varied with different ultrasonic frequency treatments. An appropriate frequency model could increase the content of SPs. Similar results were observed in the study of Alenyorege et al. (31), in which treatment with 28 ± 2 kHz increased the content of SPs in the Chinese cabbages while treatment with 28/68 kHz could decrease the content of SPs. Additionally, the content of IPs (approximately 3–5 mg GAE/100 g FW) in fresh-cut red cabbages was much lower than the content of SPs. Moreover, there was no significant difference in the content of IPs after ultrasonic washing during storage compared with control (*P* > 0.05), whatever the frequency mode was. These results showed that the increase in the content of SPs in fresh-cut red cabbages was irrelevant to the IPs, although some researchers found that the increase in the content of SPs in extracts after ultrasound treatment could be due to the breakage of cross-links in IPs (41). Therefore, the subsequent analysis of the effects of ultrasonic washing on phenolics in fresh-cut red cabbages will be conducted from the perspective of SPs.

In order to compare the effects of various frequency treatments on the content of SPs, the retention rate of SPs under the treatments of single-fixed, single-sweep, dual-fixed, and dual-sweep frequency are summarized in Supplementary Figure 3. As observed from Supplementary Figures 3A,B, on the whole, the phenolic retention rate after single-sweep frequency treatments was higher than that treated by single-fixed frequency. Although fresh-cut red cabbages treated by 33 ± 2 kHz showed the highest phenolic retention rate on Day 2, the phenolics were unstable during the following storage and markedly degraded. Meanwhile, the phenolic retention rate in fresh-cut red cabbages treated by 28 ± 2 kHz remained high level among the whole storage and increased to the maximum on Day 6. For the dual-sweep frequency treatment (as seen in Supplementary Figures 3C,D), the 40/68 kHz treatment was more conducive to the retention of phenolics than the other combined treatments.

**FIGURE 2:**
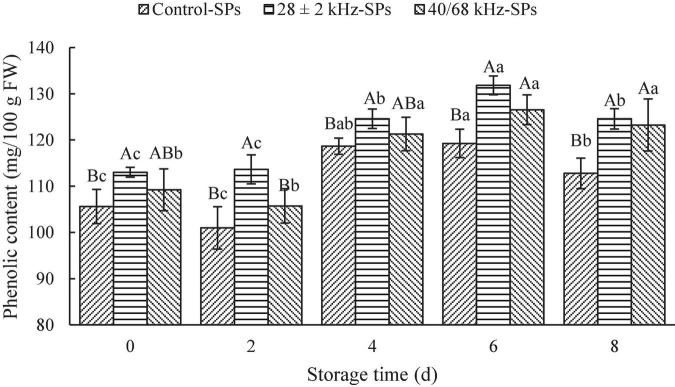
Effects of the optimal single-frequency and dual-frequency mode on the content of soluble phenolics (SPs) in fresh-cut red cabbages. The results represent the means of three triplicates ± standard deviations. Different capital letters mean that the effects of different treatments for the same day are significantly different (*P* < 0.05); different lowercase letters mean that the effects of storage times for the same treatment are significantly different (*P* < 0.05). Control: fresh-cut red cabbages were washed with sterile distilled water; treatment: ultrasonic power density of 60 W/L, frequency cycle time of 500 ms, and ultrasonic time of 15 min.

The effects of the optimal single-frequency (28 ± 2 kHz) and dual-frequency (40/68 kHz) mode on the content of SPs in fresh-cut red cabbages are shown in Figure 2. The content of SPs in fresh-cut red cabbages washed with 28 ± 2 kHz was significantly increased compared with the control (*P* < 0.05), and the samples treated with 28 ± 2 kHz (113.64 ± 3.14 mg GAE/100 g FW) showed higher content of SPs than that washed with 40/68 kHz (105.73 ± 3.71 mg GAE/100 g FW) on Day 2. Therefore, the 28 ± 2 kHz treatment was more suitable for fresh-cut red cabbages.

#### Effects of frequency amplitude on the content of soluble phenolics

**FIGURE 3:**
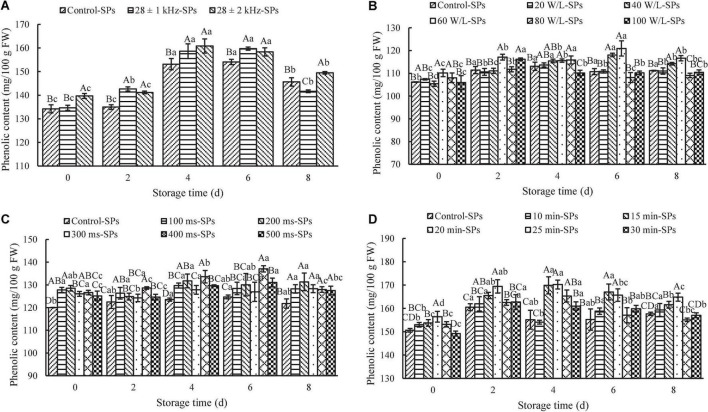
Effects of ultrasonic power density, frequency amplitude, frequency cycle time, and ultrasonic time on the content of soluble phenolics (SPs). The results represent the means of three triplicates ± standard deviations. Different capital letters mean that the effects of different treatments for the same day are significantly different (*P* < 0.05); different lowercase letters mean that the effects of storage times for the same treatment are significantly different (*P* < 0.05). **(A)** Frequency amplitude; treatment: ultrasonic power density of 60 W/L, frequency cycle time of 500 ms, and ultrasonic time of 15 min. **(B)** Ultrasonic power density; treatment: ultrasonic frequency mode of 28 ± 2 kHz, frequency cycle time of 500 ms, and ultrasonic time of 15 min. **(C)** Frequency cycle time; treatment: ultrasonic frequency mode of 28 ± 2 kHz, ultrasonic power density of 60 W/L, and ultrasonic time of 15 min. **(D)** Ultrasonic time; treatment: ultrasonic frequency mode of 28 ± 2 kHz, ultrasonic power density of 60 W/L, and frequency cycle time of 400 ms. Control: fresh-cut red cabbages were washed with sterile distilled water.

The effects of frequency amplitude on the content of SPs in fresh-cut red cabbages are shown in Figure 3A. The 28 ± 2 kHz treatment (139.68 ± 1.06 mg GAE/100 g FW) significantly increased the content of SPs compared with the 28 ± 1 kHz treatment (134.65 ± 1.08 mg GAE/100 g FW) and control (134.22 ± 1.71 mg GAE/100 g FW) on Day 0 (*P* < 0.05), while there was no significant difference between the 28 ± 1 kHz treatment and control. Moreover, the content of SPs treated at 28 ± 2 kHz and 28 ± 1 kHz was significantly higher than the control during the following storage (*P* < 0.05), except for a decrease under the 28 ± 1 kHz treatment on Day 8. These results suggested that ±2 kHz was more conducive to the accumulation of phenolics in fresh-cut red cabbages. Frequency amplitude is one of the important factors that affect the quality of fruit and vegetables during the ultrasonic washing process (32). Wang et al. (42) also found that frequency amplitude could affect the stability of caffeic acid and erucic acid.

#### Effects of ultrasonic power density on the content of soluble phenolics

The effects of ultrasonic power density on the content of SPs in fresh-cut red cabbages are displayed in Figure 3B. The 60-W/L treatment showed the highest content of SPs (120.93 ± 3.34 mg GAE/100 g FW) compared with other treatments during the whole storage. Meanwhile, it can be found that excessively low or high power density was harmful to the accumulation of SPs in fresh-cut red cabbage. The content of SPs in fresh-cut red cabbages treated with a low power density (e.g., 20 W/L) showed no significant difference compared with the control during storage (*P* > 0.05). Meanwhile, high power density (e.g., 80 and 100 W/L) was not conducive to the retention of phenolics during the later storage. Similar results were also found in Pinheiro et al. (43), Wang et al. (44), and Wu et al. (45). For example, Wang et al. (44) found that there was no significant difference in the content of SPs in cherry tomatoes between the 66.4 W/L treatment and control during storage (*P* > 0.05). Tomatoes treated with 145.79 W/L had lower content of SPs than the control during the later storage, while the content of SPs under the treatment of 106.19 W/L showed an increase during the later storage, and it was significantly higher than that in the control group on Day 20 (*P* < 0.05). Wu et al. (45) also found that the content of SPs in bok choy under the 150-W treatment and 180-W treatment significantly increased compared with the control (*P* < 0.05), while the 120-W treatment and 210-W treatment had no significant difference compared with the control (*P* > 0.05). Based on the results above, the ultrasonic power density of 60 W/L was selected as the optimal for further study.

#### Effects of frequency cycle time on the content of soluble phenolics

The frequency cycle time is one of the unique ultrasonic parameters in the sweep-frequency mode (32). The effects of frequency cycle time on the content of SPs in fresh-cut red cabbages are shown in Figure 3C. As can be seen from the figure, the frequency cycle time could affect the content of SPs, and an increase in the content of SPs can be observed for samples treated with different frequency cycle times compared to the control during the whole storage. The content of SPs at a short frequency cycle time (e.g., 100 and 200 ms) was remarkably higher than that at a long one (e.g., 500 ms) on Day 0. It may be due to the fact that a shorter sweep cycle means a faster change of sweep frequency, which produces a bigger vibration and strengthens the cavitation effects (42). Thus, the degree of hydroxylation in food materials was enhanced. However, the content of SPs at 400 ms markedly increased during the following storage. It reached the maximum value (137.06 ± 1.38 mg GAE/100 g FW) on Day 6, which was significantly higher than other treatments (*P* < 0.05). Based on the results above, the frequency cycle time of 400 ms was selected as the optimal for further study.

#### Effects of ultrasonic time on the content of soluble phenolics

The effects of ultrasonic time on the content of SPs in fresh-cut red cabbages are shown in Figure 3D. The content of SPs in ultrasound-treated samples increased with ultrasonic time until reaching the maximum at 20 min (156.42 ± 2.30 mg GAE/100 g FW) and then decreased with longer ultrasonic time on Day 0. The content of SPs under 30 min treatment was even lower than that of the control. On the one hand, the decrease in the content of SPs might result from the greater disruption of cell wall material and more inordinateness of inner structure caused by longer ultrasound treatment (46), leading to SPs draining away with the water during the process of ultrasonic washing. On the other hand, oxidation reactions promoted by the interaction with free radicals formed during sonication may be another reason for the degradation of phenolics (43). Additionally, the content of SPs in fresh-cut red cabbages treated for 20 min was the highest compared with other treatments throughout the storage. It firstly increased to the maximum on Day 4 and then decreased afterward. Moreover, it can be found that excessively short or long ultrasonic time was not conducive to the retention of the content of SPs in fresh-cut red cabbages. The content of SPs treated for 10 min had no significant difference compared with the control (*P* > 0.05), while the content of SPs after 30 min treatment was significantly lower than that of the 20 min treatment during storage (*P* < 0.05). Similar results were observed in the study of Lu et al. (47) and Gani et al. (48). For example, Gani et al. (48) found a significant increase in the content of SPs in strawberries as the ultrasonic time increased from 0 to 40 min during storage, followed by a decrease when the treatment time was increased to 60 min. Based on the results above, the optimal ultrasound treatment time was selected as 20 min for further study.

#### Effects of ultrasonic washing on the content of soluble phenolics

Based on the above results, it was found that appropriate ultrasonic washing could increase the content of SPs in fresh-cut red cabbages immediately or during storage. These results were consistent with the study of Yildiz et al. (24), Mustapha et al. (49), and Tovar-Pérez et al. (50). For example, Mustapha et al. (49) found that the SPs in ultrasound-treated cherry tomatoes increased with the prolongation of the storage period and was significantly higher than that in water-washed samples on Day 0 and Day 21 (*P* < 0.05). The immediate increase in the content of SPs after treatment mainly might be due to the increased extractability of phenolics, which may result from the hydroxylation of food materials caused by cavitation effects, especially flavonoids (31, 51, 52). Ashokkumar et al. (53) also reported that the hydroxylation of phenol and cyanidin 3-glucoside was initiated when treated by ultrasound. Meanwhile, as shown in Supplementary Figures 4, 5A,B, the scanning electron micrograph of cross sections in the leaves showed that the fresh-cut red cabbages treated by optimized ultrasonic conditions showed no damage to the structural anatomy of the leaves compared to the control on Day 0. The undamaged leaf structure contributes to the retention of phenolics (46). The accumulation of phenolics in fresh-cut red cabbages during storage may be attributed to the fact that the biosynthesis rate of phenolics is higher than the consumption rate (22, 54, 55). On the one hand, the biosynthetic pathway of phenolics can be activated. It is widely acknowledged that plants subjected to postharvest abiotic stresses can undergo a series of events, resulting in the accumulation of molecules with health-promoting properties, such as phenolics, carotenoids, AA, and glucosinolates (13, 14, 25, 56). When fruit and vegetables suffer from appropriate ultrasound treatment, wounded cells release adenosine triphosphate from the cytoplasm, which binds to unwounded cell receptors. The binding can elicit the production of secondary signaling molecules, such as reactive oxygen species (ROS), ethylene, and jasmonic acid. These secondary signals can activate the expression of primary and secondary metabolic genes, thus triggering the biosynthesis of phenolics (14, 56, 57). Additionally, the balance of ROS in fruit and vegetables can also be broken due to a large number of free radicals caused by acoustic cavitation (58). Therefore, the ROS scavenging systems in fruit and vegetables can be activated after ultrasonic washing. Phenolics, as one of the main components of non-enzymatic antioxidant systems, can be biosynthesized to eliminate excess ROS. It is because phenolics have dynamic antioxidant properties which neutralize the consequences produced by oxidative stress (59, 60). On the other hand, the consumption rate of phenolics after ultrasonic washing may be inhibited. Firstly, fewer phenolics can be used for lignin biosynthesis after ultrasonic washing. Phenolics are considered the precursors for lignin biosynthesis (22). Hence, the reduction of lignin content means less loss of phenolics (54). For example, Wang and Fan (61) reported that the biosynthesis of lignin in green asparagus subjected to ultrasonic washing was effectively retarded compared to the untreated ones. The delay of lignin biosynthesis was conducive to the retention of phenolics. Secondly, the activities of enzymes involved in the oxidative degradation of phenolics subjected to ultrasound treatment can be inhibited, such as polyphenol oxidase (PPO) and peroxidase (POD) (62). However, it was also found that inappropriate ultrasonic washing after fresh-cutting could decrease the content of SPs of red cabbages immediately and even reduce the cutting-induced accumulation of phenolics during storage. Similar results were found in the study of Cuéllar-Villarreal et al. (28), in which the level of total phenolics in ultrasound-treated carrots (300 s) was significantly lower than that in the control ones. It is likely that phenolics are released from the food matrix into the water due to ultrasound-induced damage to tissues (63). Additionally, the cutting-stress signals that induce the biosynthesis of phenolics may be removed by ultrasonic washing, causing the reduction of the cutting-induced accumulation of phenolics (64).

### Analysis of the activities of phenylalanine ammonia-lyase, polyphenol oxidase, and peroxidase

**FIGURE 4:**
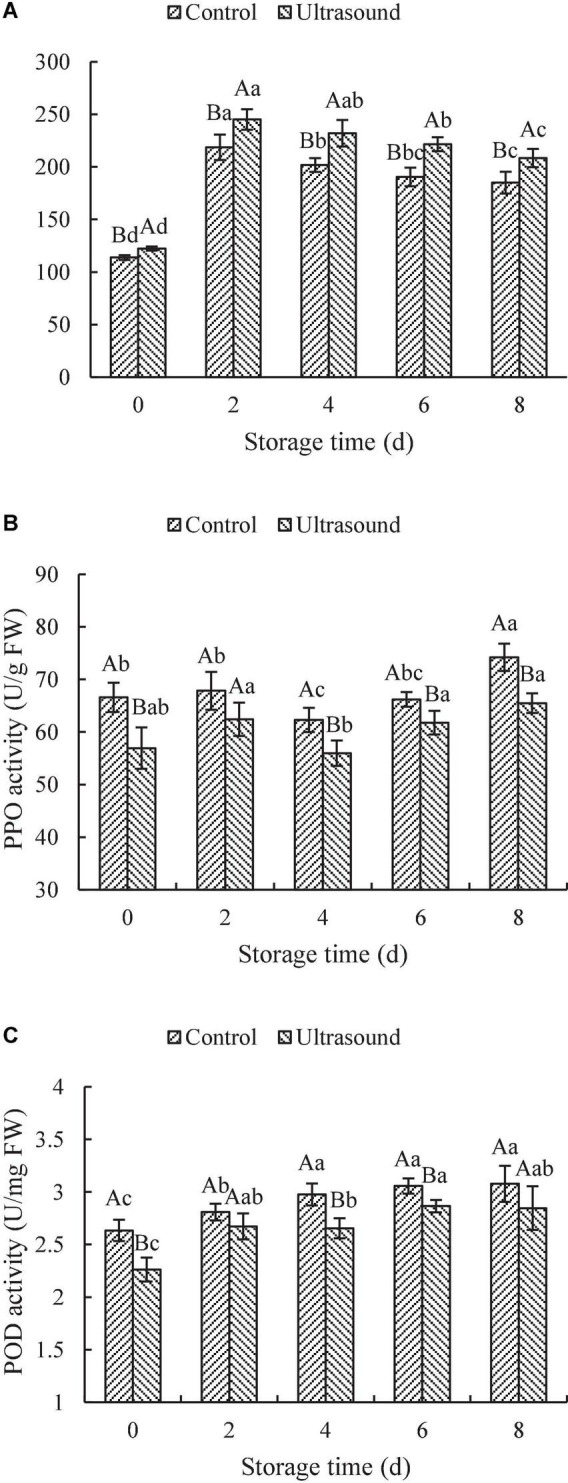
Effects of ultrasonic washing on the activities of phenylalanine ammonia-lyase (PAL) **(A)**, polyphenol oxidase (PPO) **(B)**, and peroxidase (POD) **(C)**. The results represent the means of three triplicates ± standard deviations. Different capital letters mean that the effects of different treatments for the same day are significantly different (*P* < 0.05); different lowercase letters mean that the effects of storage times for the same treatment are significantly different (*P* < 0.05). Control: fresh-cut red cabbages were washed with sterile distilled water; ultrasound treatment: ultrasonic frequency mode of 28 ± 2 kHz, ultrasonic power density of 60 W/L, frequency cycle time of 400 ms, and ultrasonic time of 20 min.

The changes in the activities of PAL, PPO, and POD in fresh-cut red cabbages treated by ultrasonic washing are shown in Figure 4. As shown in Figure 4A, the PAL activity of fresh-cut red cabbages with or without ultrasonic washing showed the tendency to first rise and then decline with the extension of the shelf-life, reaching the maximums on Day 2. In addition, the PAL activity of fresh-cut red cabbages after ultrasound treatment increased significantly compared with the control (*P* < 0.05). PAL plays a critical role in the phenylpropanoid cycle, which contributes to the conversion of *L*-phenylalanine to trans-cinnamic acid with ammonia elimination and induces the biosynthesis of various phenylpropanoid-derived secondary products, such as SPs (65). Therefore, the increase in PAL activity is conducive to the biosynthesis of phenolics. The results shown in Figure 4A and Figure 3D indicated that the change trends of PAL and SPs were similar. However, the changes in the content of SPs performed a more noticeable lag effect than PAL changes. Similar lag effects were also observed in the study of Zhu et al. (66), in which they found that the changes in the SPs of fresh-cut potato slices at different ultrasound treatments were similar to the changes in the PAL activity and were delayed to a certain extent. PAL has been considered an inducible enzyme under biotic and abiotic stress (65). Therefore, cutting may be responsible for the increase of PAL activity of fresh-cut red cabbages in early storage. For example, Heredia and Cisneros-Zevallos (67) found that cutting could induce the activity of PAL in carrots and the PAL activity increased with wounding intensity. Whereas the decrease in PAL activity during the later storage may be due to aging and browning consumption till the end (68). Additionally, PAL activity has also been proposed to play a significant role in the plant defense against ultrasound stress, such as fresh-cut pineapples, Roman lettuces, carrots, Lentinula edodes, and cherry tomatoes (27, 28, 69–71). It might be because the formation of free radicals due to the sonolysis of water may impose oxidative stress on the plant cell systems and induce higher PAL activity to protect the cells from oxidative damage (27). Therefore, the PAL activity and the content of SPs in ultrasound-treated fresh-cut red cabbages were induced at higher levels under the combined mechanical and oxidative stress injury during storage.

According to Figure 4B, the PPO activity after ultrasonic washing was significantly lower than that in control samples during the whole storage (*P* < 0.05), except on Day 2. Similarly, the POD activity in control samples was also relatively higher than that in ultrasound-treated ones throughout the cold storage (as shown in Figure 4C). The POD activity in fresh-cut red cabbages with or without ultrasonic washing increased firstly and then remained stable with the extension of storage time. The POD activity during the later stage (e.g., on Day 6 and Day 8) significantly increased compared with that on Day 0, which may be related to tissue senescence (44, 72). Based on the results above, it was found that both the PPO and POD activities in fresh-cut red cabbages subjected to ultrasonic washing could be inhibited throughout the cold storage. Similar results were found in the study of Yeoh and Ali (27) and Pan et al. (68). For example, Yeoh and Ali (27) demonstrated that PPO and POD activities in fresh-cut pineapples were suppressed by ultrasound treatments, which supported our results. Thus, the browning inhibition of fresh-cut red cabbages after ultrasound treatment was exerted by reducing the activity of PPO and POD, which were typical enzymes involved in browning. It is universally acknowledged that phenolics can act as hydrogen donors to participate in the decomposition reaction of hydrogen peroxide catalyzed by POD, and PPO can convert phenolics into quinones (65). The phenolic content in fruit and vegetables correlates with the PPO and POD activities, and the higher levels of PPO and POD activities can cause more consumption of phenolic content. Therefore, the decrease in the content of SPs in fresh-cut red cabbages during the later storage may result from high levels of PPO and POD activities. Meanwhile, the consumption of SPs of fresh-cut red cabbages after ultrasonic washing was inhibited due to the inhibition of PPO and POD activities, which consequently resulted in higher content of SPs in ultrasound-treated fresh-cut red cabbages in comparison to control. The physiological enzyme activity can be activated and passivated by ultrasound treatment. The enzyme inhibition effect of ultrasound treatment depends on the chemical structure of proteins and the tolerance of enzymes to ultrasound (73). The inhibition of PPO and POD activities after ultrasound treatment may result from the physical and chemical effects of cavitation, which produce shear forces to break down the Van der Waals forces and hydrogen bonding of polypeptide chains, leading to the modification of enzyme structures (74).

### Analysis of quality parameters

#### Effects of ultrasonic washing on weight retention rate and respiration rate

**FIGURE 5:**
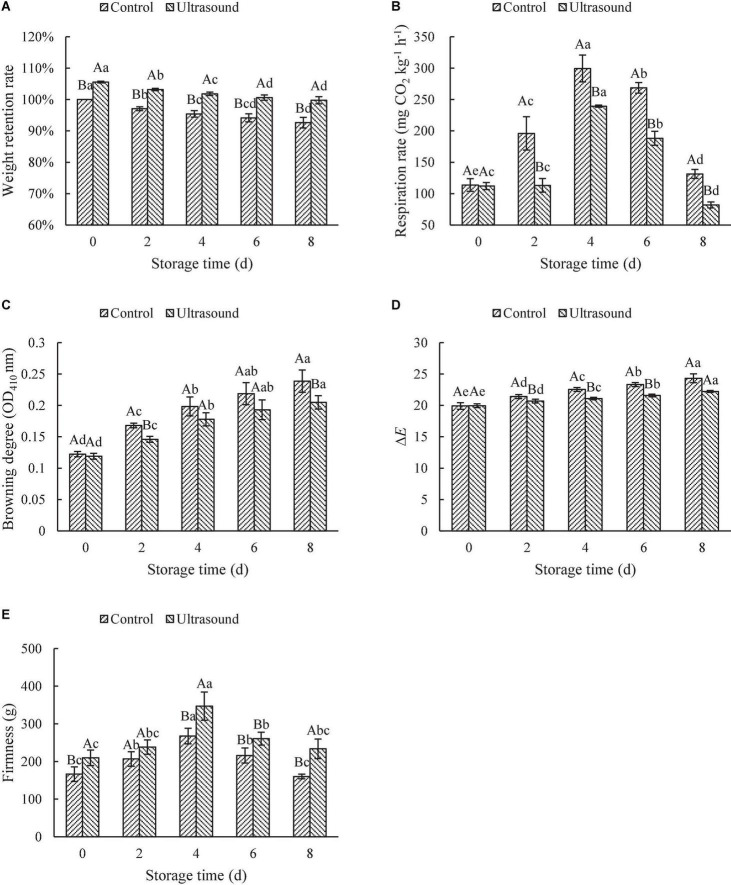
Effects of ultrasonic washing on the weight retention rate **(A)**, respiration rate **(B)**, color **(C,D)**, and firmness **(E)**. The results represent the means of three triplicates ± standard deviations. Different capital letters mean that the effects of different treatments for the same day are significantly different (*P* < 0.05); different lowercase letters mean that the effects of storage times for the same treatment are significantly different (*P* < 0.05). Control: fresh-cut red cabbages were washed with sterile distilled water; ultrasound treatment: ultrasonic frequency mode of 28 ± 2 kHz, ultrasonic power density of 60 W/L, frequency cycle time of 400 ms, and ultrasonic time of 20 min.

The changes in the weight of ultrasound-treated fresh-cut red cabbages during storage are shown in Figure 5A. Weight is considered an important indicator in evaluating the quality of postharvest fruit and vegetables, and its reduction reflects water loss during storage (75). As seen in Figure 5A, the weight of fresh-cut red cabbages increased significantly after ultrasonic washing (*P* < 0.05). It is probably because the mass transfer of water from the liquid medium to the samples can be promoted by ultrasound. Similar results were also observed in the study of Wang et al. (76), in which carrot slices immersed in distilled water gained more water after processing with low-frequency ultrasound treatment than untreated samples. Moreover, as shown in Figure 5A, the weight retention rate of fresh-cut red cabbages after ultrasonic washing was significantly higher than that of control during the whole storage, although the weight of fresh-cut red cabbage decreased with the increase of storage time (*P* < 0.05). Lower weight loss was observed in the ultrasound-treated samples. The epidermal cells in the leaves of fresh-cut red cabbages had higher water content compared with that in control ones (as shown in Supplementary Figure 5). It might be because the water molecules were confined by hydrogen bonds, which decreased the loss of water (36). Similarly, Fan et al. (77) also found that the water loss of fresh-cut lettuces after ultrasonic washing was reduced compared with the control.

Respiration rate is an important parameter in determining deterioration rate and the onset of senescence, which is proportional to product deterioration rate and inversely proportional to its shelf-life. As shown in Figure 5B, the respiratory rate of fresh-cut red cabbages with or without ultrasonic washing increased firstly and then decreased during storage, reaching the maximum on Day 4. The increase in the respiratory rate at the initial storage stage might be due to the cutting (mechanical damages), which boosted the respiratory rate (78). Whereas the decrease of it during the later storage may result from the large consumption of materials in the early storage (e.g., sugars and O_2_), which inhibited the respiration rate. Li et al. (36) also found that the activities of enzymes involved in respiration (e.g., succinic dehydrogenase, glucose-6-phosphate dehydrogenase, and 6-phosphogluconate dehydrogenase) increased firstly and then decreased with the extension of storage time, which may be another other reason for the changes in the respiration rate. Additionally, the respiration rate after ultrasonic washing was significantly lower than that of the control (*P* < 0.05). Similar results were also found in other vegetables subjected to ultrasonic washing, such as lettuces, white mushrooms, straw mushrooms, and asparaguses (36, 74, 79, 80). On the one hand, the lower respiration rate can be attributed to the hydrogen peroxide formation in distilled water during sonication, which reduces the oxygen used for respiration (81). On the other hand, ultrasonic washing can decrease the activities of respiration enzymes, thus reducing the respiration rate (36). Li et al. (36) found that ultrasound treatment could inhibit respiratory rates *via* inactivating the activities of enzymes involved in respiratory pathways of straw mushrooms, including phosphohexoisomerase, succinic dehydrogenase, glucose-6-phosphate dehydrogenase, 6-phosphogluconate dehydrogenase, and cytochrome oxidase. Moreover, more stomata in the leaves of fresh-cut red cabbages after ultrasonic washing were closed since epidermal cells with high water content squeezed the guard cells (as shown in Supplementary Figure 5). The closure of stomata reduced the respiration rate of fresh-cut red cabbages, which may also contribute to the retention of water during storage. Therefore, ultrasound treatment could effectively inhibit the respiration rate of fruit and vegetables to improve storage quality. However, it was found that ultrasound could increase the respiration rate in previous literature (28). Inconsistent results could be explained by the differences in ultrasonic conditions (e.g., frequency, time, and power output) and types of fruit and vegetables (30, 44).

#### Effects of ultrasonic washing on color and firmness

Color is one of the most important factors that reflect the appearance quality of fresh-cut fruit and vegetables, which directly affects the purchase intention of the consumers. Browning is a particular problem for white-fleshed fresh-cut products including red cabbages due to the oxidations of phenolics triggered by polyphenol oxidase (39). The effects of ultrasonic washing on the browning degree and total color difference (Δ*E*) of fresh-cut red cabbages during storage are shown in Figures 5C,D. There was no significant difference in browning degree and Δ*E* between the ultrasound and control samples on Day 0. Then the browning degree and Δ*E* with or without ultrasonic washing showed an increasing trend with the extension of storage time, and the fresh-cut red cabbages treated by ultrasound showed a significantly lower browning degree and Δ*E* values compared with the control (*P* < 0.05). The results suggested that ultrasonic washing could effectively inhibit the browning of fresh-cut red cabbages during storage and maintain the appearance of the color (as shown in Supplementary Figure 6). These results were consistent with the conclusions of Li et al. (36), Yildiz et al. (39), and Wen et al. (82). For example, Wen et al. (82) observed a noticeable increase in whiteness index, a decrease in the browning index, and lower Δ*E* values in ultrasound-treated fresh-cut lotus roots compared to the control during storage. These phenomena might result from the changes in PPO and POD activity related to the browning (36). Qiao et al. (29) also found that the PPO and POD activity in fresh-cut potatoes were inhibited by ultrasound washing, thus alleviating the enzymatic browning during storage.

Firmness is an important determinant of textural quality that affects consumer preference and the acceptability of fresh-cut fruit and vegetables. The changes in firmness depend on the cell turgor pressure, the integrity of the cell wall, and intercellular adhesion (12). As shown in Figure 5E, the firmness of fresh-cut red cabbages with or without ultrasonic washing rose to the maximum on Day 4, followed by a decrease with the increase in storage time. Fresh-cut red cabbages treated by ultrasound exhibited higher firmness than the control during storage. Similarly, Gani et al. (48) also found that the firmness of strawberries treated by ultrasonic washing for 10–40 min rose firstly and then declined with the increasing storage time. Fruit firmness under ultrasound treatment was higher than untreated samples throughout all refrigerated storage. Likewise, Yu et al. (69) also observed that lettuces treated by ultrasound showed higher firmness than the water-washed ones during storage, and the firmness increased with the storage time. The optimized ultrasound treatment did not affect the structural anatomy of the leaves as mentioned before, which was beneficial to maintain the texture characteristics. The increase in firmness may be due to the fact that the self-recovery system of plants was initiated when treated with ultrasound and the production of phenolic compounds might help the plants regain tissue firmness (69). Additionally, another possible reason is that ultrasound treatment inhibits the activities of enzymes that are mainly responsible for fruit softening, such as pectin methylesterase and polygalacturonase (83). However, the loss of firmness at the later stage of storage is most often attributed to the enzymatic breakdown of the middle lamella and cell wall by pectin methylesterase, polygalacturonase, β-galactosidase, and cellulase (84). Moreover, the water of leaves also influences the firmness of fruit and vegetables. Softening of fruit and vegetables is relatively attributed to a considerable increase in water loss through respiration (85, 86). The loss of turgor of the cell (loss of water) can produce dehydration of the tissues, an increase in elasticity, and a decrease in firmness (87), especially at the end of the storage (as shown in Supplementary Figures 5E,F). However, further studies are needed to monitor the moisture loss of fruit and vegetables subjected to ultrasonic washing and its relation to texture characteristics during storage.

#### Effects of ultrasonic washing on the contents of ascorbic acid, total soluble proteins, total soluble sugars, and total soluble solids

The AA content is an important index to evaluate the nutritional value of fruit and vegetables (36). As shown in Table 2, although the AA content of fresh-cut red cabbages after ultrasonic washing was higher than that of the control ones, there was only a significant difference on Day 4 during storage (*P* < 0.05). Similarly, Wang et al. (44) also found that the AA content of cherry tomatoes treated with ultrasonic washing at 106.19 and 145.74 W/L was significantly higher than that of the control on Day 8 or Day 16 (*P* < 0.05), while there was no significant difference during other storage time. The higher retention rate of AA content in fruit and vegetables after ultrasonic washing can be attributed to the removal of dissolved oxygen caused by the cavitation effects of ultrasound, which inhibited the oxidation of AA (23, 61). Furthermore, the inactivation of fruit enzymes induced by ultrasound treatment, e.g., AA oxidase and lipoxygenase, maybe another reason to prevent the degradation of AA (11). However, it is also found that ultrasonic washing has no effects on AA content in some fruit and vegetables, and sometimes even has negative effects (88). Overall, current reports regarding the effects of ultrasound on AA content in fruit and vegetables have been controversial. These differences could be attributed to variations in species, treatments, and analytical methods.

**TABLE 2:** Effects of ultrasonic washing on the contents of ascorbic acid (AA), total soluble proteins (TSPs), total soluble sugars (TSSs), and total soluble solids (SSs) in fresh-cut red cabbages during 4°C storage.

Nutrient	Treatment	Storage time				
		
		Day 0	Day 2	Day 4	Day 6	Day 8
AA (mg/100 g FW)	Control	83.16 ± 2.29^Ac^	85.97 ± 3.69^Abc^	91.38 ± 3.19^Ba^	89.62 ± 3.65^Aab^	89.61 ± 2.37^Aab^
	Ultrasound	85.99 ± 1.92^Ad^	88.61 ± 2.74^Acd^	99.46 ± 3.17^Aa^	93.21 ± 3.28^Ab^	92.58 ± 4.09^Abc^
TSPs (mg/g FW)	Control	2.07 ± 0.21^Ac^	2.45 ± 0.06^Ab^	2.24 ± 0.25^Bbc^	2.88 ± 0.12^Ba^	2.78 ± 0.15^Aa^
	Ultrasound	2.09 ± 0.16^Ad^	2.44 ± 0.08^Ac^	2.59 ± 0.12^Ac^	3.41 ± 0.11^Aa^	3.02 ± 0.21^Ab^
TSSs (g/g FW)	Control	30.31 ± 1.05^Aa^	27.49 ± 1.19^Ab^	24.15 ± 1.16^Ac^	22.70 ± 0.87^Bc^	22.68 ± 1.59^Bc^
	Ultrasound	30.85 ± 1.45^Aa^	28.01 ± 1.14^Ab^	25.75 ± 1.03^Ac^	24.99 ± 1.15^Ac^	25.08 ± 0.97^Ac^
SSs (%)	Control	0.50 ± 0.02^Aa^	0.48 ± 0.02^Ba^	0.50 ± 0.01^Aa^	0.34 ± 0.03^Bc^	0.30 ± 0.01^Bd^
	Ultrasound	0.49 ± 0.03^Ab^	0.53 ± 0.02^Aa^	0.52 ± 0.02^Aab^	0.45 ± 0.03^Ac^	0.40 ± 0.02^Ad^

The results represent the means of three triplicates ± standard deviations. Different capital letters mean that the effects of different treatments for the same day are significantly different (*P* < 0.05); different lowercase letters mean that the effects of storage times for the same treatment are significantly different (*P* < 0.05). Control: fresh-cut red cabbages were washed with sterile distilled water; ultrasound: ultrasonic frequency mode of 28 ± 2 kHz, ultrasonic power density of 60 W/L, frequency cycle time of 400 ms, and ultrasonic time of 20 min. FW, fresh weight.

Proteins are considered nutrient sources to support the physiological metabolisms of fruit and vegetables and are important to the evaluation of nutrition and quality. As shown in Table 2, the content of TSPs in fresh-cut red cabbages subjected to ultrasonic washing was higher than that of the control during the later storage, especially on Day 4 and Day 6. Similar results were also observed in the study of Li et al. (36), in which 10-min ultrasonic washing could effectively prevent the utilization of TSPs in straw mushrooms, thus retaining more TSPs compared with the control. Therefore, ultrasonic washing can contribute to the retention of proteins in fruit and vegetables. However, there are rare reports on the mechanisms that explain the effects of ultrasound on the proteins of fruit and vegetables. More retention of TSPs after ultrasound treatment can be explained by the inhibition of enzymes related to protein degradation, such as succinic dehydrogenase and lipoxygenase (11, 36). Consequently, the oxidation and catabolism of amino acids were suppressed, improving the retention.

Sugars are important energy sources of fruit and vegetables, mainly involved in the carbohydrate metabolisms in cells. Effects of ultrasonic washing on the content of TSSs are shown in Table 2. The content of TSSs in fresh-cut red cabbages with or without ultrasonic washing declined gradually with the prolonged storage time. At the end of the storage time, fresh-cut red cabbages treated with ultrasound showed higher content of TSSs compared with the control. Similarly, Li et al. (36) also found that ultrasound treatment could notably retain the content of TSSs in straw mushrooms and reduce their consumption. Therefore, the degradation of TSSs in fruit and vegetables can be alleviated after ultrasonic washing, which is beneficial to the maintenance of quality. The consumption of sugars is generally related to normal metabolic processes, especially respiratory rate (85, 89, 90). It can be found that more TSSs of fresh-cut red cabbages were consumed with the increase in the respiratory rate at the beginning of the storage time. The consumption of TSSs was reduced when the respiratory rate of samples was inhibited with ultrasound. Moreover, Fan et al. (75) thought that the activity of enzymes related to carbohydrate metabolisms could be inhibited by the cavitation effects of ultrasound, causing more reservations of sugars. Similarly, Li et al. (36) also found that the activities of enzymes involved in the glycolytic pathway and hexose monophosphate pathway of straw mushrooms were inhibited by ultrasound treatment, including phosphohexoisomerase, glucose-6-phosphate dehydrogenase, and 6-phosphogluconate dehydrogenase.

The changes in the content of SSs in fresh-cut red cabbages after ultrasonic washing are shown in Table 2. The content of SSs in ultrasound-treated samples increased firstly and then decreased with the prolonged storage time, and it was significantly higher than that in the control ones at the end of storage time. Similar results were also found in the study of Temizkan et al. (90), in which the content of SSs in white nectarines treated with ultrasound at 300 W increased firstly until reaching the maximum on Day 5 and then decreased, and the values were significantly higher than the control ones during the storage time from Day 10 to Day 45. Higher content of SSs may be associated with lower metabolisms, i.e., reduced respiration and delayed senescence (91). The decrease in the content of SSs during the later storage may result from the consumption of respiration (90). In addition, Wang and Fan (61) also thought that the high retention of SSs in green asparaguses subjected to ultrasonic washing was due to the inactivation of enzymes related to metabolisms caused by ultrasound treatment. The activities of enzymes mentioned above involved in respiration and carbohydrate metabolisms were suppressed, thus reducing the consumption of SSs.

### Analysis of microbial safety

Cutting can increase the contact area between the microorganisms and fruit and vegetables. Meanwhile, the juice flowing from shredded tissues can provide favorable breeding conditions for microorganisms, increasing food corruption and quality deterioration (8, 9, 92). The effects of ultrasonic washing on the microorganisms in fresh-cut red cabbages during storage are shown in Table 3. At the initial storage stage, fresh-cut red cabbages with or without ultrasonic washing showed a low total number of bacteria, molds and yeasts, and then they presented a rapid growth with the increase in storage time. However, the microbial numbers after ultrasonic washing were significantly lower than the control during storage (*P* < 0.05). The total number of bacteria, molds and yeasts in ultrasound samples on Day 8 (4.57 ± 0.05 and 2.84 ± 0.05 log_10_ CFU/g, respectively) were even lower than the control ones on Day 6 (4.95 ± 0.03 and 3.16 ± 0.02 log_10_ CFU/g, respectively). The decrease in the number of microorganisms might be due to the bactericidal effects and shock effects of ultrasound (93). Microorganisms can be inactivated by cavitation effects to some extent due to high temperature, high pressure, and free radicals produced by bubble explosion (21). Meanwhile, powerful jets produced by the collapse of cavitation bubbles would dislodge microorganism cells on the fruit surface (83). All of these can reduce the number of microorganisms after ultrasonic washing. Similar phenomena have been observed in cucumbers, bok choy, lettuces, kiwifruit, and quince (24, 39, 45, 75, 77). Therefore, ultrasonic washing can slow down the growth of microorganisms and reduce the decay incidence of fresh-cut red cabbages.

**TABLE 3:** Effects of ultrasonic washing on the microorganisms of fresh-cut red cabbages during 4°C storage.

Microorganism	Treatment	The number of microorganisms during storage (log_10_ CFU/g)				
		
		Day 0	Day 2	Day 4	Day 6	Day 8
Bacteria	Control	2.57 ± 0.03^Ae^	3.23 ± 0.01^Ad^	4.01 ± 0.05^Ac^	4.95 ± 0.03^Ab^	5.53 ± 0.05^Aa^
	Ultrasound	2.23 ± 0.07^Be^	2.99 ± 0.01^Bd^	3.66 ± 0.05^Bc^	3.96 ± 0.03^Bb^	4.67 ± 0.05^Ba^
Molds and yeasts	Control	1.63 ± 0.05^Ae^	2.19 ± 0.02^Ad^	2.89 ± 0.05^Ac^	3.16 ± 0.02^Ab^	3.71 ± 0.03^Aa^
	Ultrasound	1.19 ± 0.06^Be^	1.94 ± 0.02^Bd^	2.12 ± 0.02^Bc^	2.64 ± 0.05^Bb^	2.84 ± 0.03^Ba^

The results represent the means of three triplicates ± standard deviations. Different capital letters mean that the effects of different treatments for the same day are significantly different (*P* < 0.05); different lowercase letters mean that the effects of storage times for the same treatment are significantly different (*P* < 0.05). Control: fresh-cut red cabbages were washed with sterile distilled water; ultrasound: ultrasonic frequency mode of 28 ± 2 kHz, ultrasonic power density of 60 W/L, frequency cycle time of 400 ms, and ultrasonic time of 20 min.

## Conclusion

In order to evaluate the feasibility of ultrasonic washing on the phenolic accumulation in fresh-cut red cabbages, the effects of ultrasound operating parameters on the phenolics were studied by single-factor tests. The optimal ultrasonic washing conditions, i.e., single-sweep frequency of 28 ± 2 kHz, power density of 60 W/L, frequency cycle time of 400 ms, and ultrasonic time of 20 min, were obtained, under which the content of soluble phenolics (SPs) was significantly increased compared with the control (*P* < 0.05). The activity of phenylalanine ammonia-lyase (PAL) related to phenolic biosynthesis after ultrasonic washing increased markedly during the whole storage, whereas the polyphenol oxidase (PPO) and peroxidase (POD) related to phenolic degradation decreased remarkably. Meanwhile, the storage quality of fresh-cut red cabbages under the optimized ultrasonic washing was improved, including reduced weight loss, decreased respiration rate, alleviated browning, and increased firmness. Besides, more AA, TSPs, TSSs, and SSs of fresh-cut red cabbages were retained during storage. Moreover, the growth of microorganisms was inhibited by ultrasonic washing, thus improving the edible safety of fresh-cut red cabbages and prolonging their shelf-life. In conclusion, ultrasonic washing could be used as an abiotic elicitor to increase the phenolics of fresh-cut red cabbages during storage and improve the storage quality and microbial safety simultaneously. Therefore, ultrasonic washing is promising to represent as an alternative sanitization step for fruit and vegetables to develop high quality and extended shelf-life of ready-to-eat fresh fruit and vegetables. However, further studies are needed to elucidate the accumulation of phenolics in fruit and vegetables caused by ultrasound stress because phenolics can be affected by various factors, such as the types of fruit and vegetables and treatment conditions. Additionally, the mechanisms underlying the enhancement of phenolic content after ultrasonic washing also need to be further explored, which is useful to regulate the defense systems of fruit and vegetables to obtain improved nutritional quality.

## Data availability statement

The original contributions presented in this study are included in the article/Supplementary material, further inquiries can be directed to the corresponding authors.

## Author contributions

CH contributed to the conceptualization, methodology, formal analysis, and writing—original draft. Y-MZ and H-CZ contributed to the writing—review and editing. HM contributed to the conceptualization, resources, and supervision. All authors contributed to the article and approved the submitted version.
