# Corrigendum: Ultrasonic washing as an abiotic elicitor to induce the accumulation of phenolics of fresh-cut red cabbages: Effects on storage quality and microbial safety

**DOI:** 10.3389/fnut.2022.1089902

**Published:** 2022-11-17

**Authors:** Chen Hong, Hong-Chang Zhou, Yi-Ming Zhao, Haile Ma

**Affiliations:** ^1^School of Food and Biological Engineering, Jiangsu University, Zhenjiang, China; ^2^Institute of Food Physical Processing, Jiangsu University, Zhenjiang, China

**Keywords:** ultrasonic washing, fresh-cut red cabbages, phenolic content, phenolic accumulation, storage quality, microbial safety

In the published article, there was an error in the Funding statement. The funding statement for the National Natural Science Foundation of China was displayed as “No. 8111360022”. The number is incorrect. The correct Funding statement appears below.

We acknowledge the financial support of the National Natural Science Foundation of China (No. 32072353) and the Senior Talent Program of Jiangsu University (No. 5501360012).

The authors apologize for this error and state that this does not change the scientific conclusions of the article in any way. The original article has been updated.

## Publisher's note

All claims expressed in this article are solely those of the authors and do not necessarily represent those of their affiliated organizations, or those of the publisher, the editors and the reviewers. Any product that may be evaluated in this article, or claim that may be made by its manufacturer, is not guaranteed or endorsed by the publisher.

