# Supplementary material for: Ultrasonic washing as an abiotic elicitor to induce the accumulation of phenolics of fresh-cut red cabbages: Effects on storage quality and microbial safety
Source: Front Nutr. 2022 Nov 3;9:1006440. doi: 10.3389/fnut.2022.1006440 (PMC9670152; doi:10.3389/fnut.2022.1006440)

# *Supplementary Material*

**Figure Captions of Supplementary Figures**

Figure S1. Effects of single-frequency mode on the phenolic content of fresh-cut red cabbages during 4 °C storage. The results represent the means of three triplicates ± standard deviations. Different capital letters mean that the effects of different treatments for the same day are significantly different ($P$ < 0.05); Different lowercase letters mean that the effects of storage times for the same treatment are significantly different ($P$ < 0.05). Control: fresh-cut red cabbages were washed with sterile distilled water; Treatment: ultrasonic power density of 60 W/L, frequency amplitude of ± 2 kHz, frequency cycle time of 500 ms, and ultrasonic time of 15 min. SPs: soluble phenolics; IPs: insoluble-bound phenolics.

Figure S2. Effects of dual-frequency mode on the phenolic content of fresh-cut red cabbages during 4 °C storage. The results represent the means of three triplicates ± standard deviations. Different capital letters mean that the effects of different treatments for the same day are significantly different ($P$ < 0.05); Different lowercase letters mean that the effects of storage times for the same treatment are significantly different ($P$ < 0.05). Control: fresh-cut red cabbages were washed with sterile distilled water; Treatment: ultrasonic power density of 60 W/L, frequency amplitude of (± 2) kHz, frequency cycle time of 500 ms, and ultrasonic time of 15 min. SPs: soluble phenolics; IPs: insoluble-bound phenolics.

Figure S3. Effects of single-frequency and dual-frequency mode on the retention rate of soluble phenolics (SPs) in fresh-cut red cabbages during 4 °C storage. The results represent the means of three triplicates ± standard deviations. (A) single-fixed frequency; (B) single-sweep frequency; (C) dual-fixed frequency; (D) dual-sweep frequency.

Figure S4. Scanning electron micrograph of cross sections in fresh-cut red cabbage leaves. A: ultrasound-treated cross section (Day 0); B: untreated cross section (Day 0).

Figure S5. Scanning electron micrograph of abaxial epidermis in fresh-cut red cabbage leaves. A, C, and E shows the image of fresh-cut red cabbages after ultrasonic washing on Day 0, Day 4, and Day 8, respectively, while B, D, and F shows the image of fresh-cut red cabbages in the control group on Day 0, Day 4, and Day 8, respectively.

Figure S6. Visual images of fresh-cut red cabbages during 4 °C storage. Control: fresh-cut red cabbages were washed with sterile distilled water; Ultrasound treatment: ultrasonic frequency mode of 28 ± 2 kHz, ultrasonic power density of 60 W/L, frequency cycle time of 400 ms, and ultrasonic time of 20 min.

**Figure S1.**

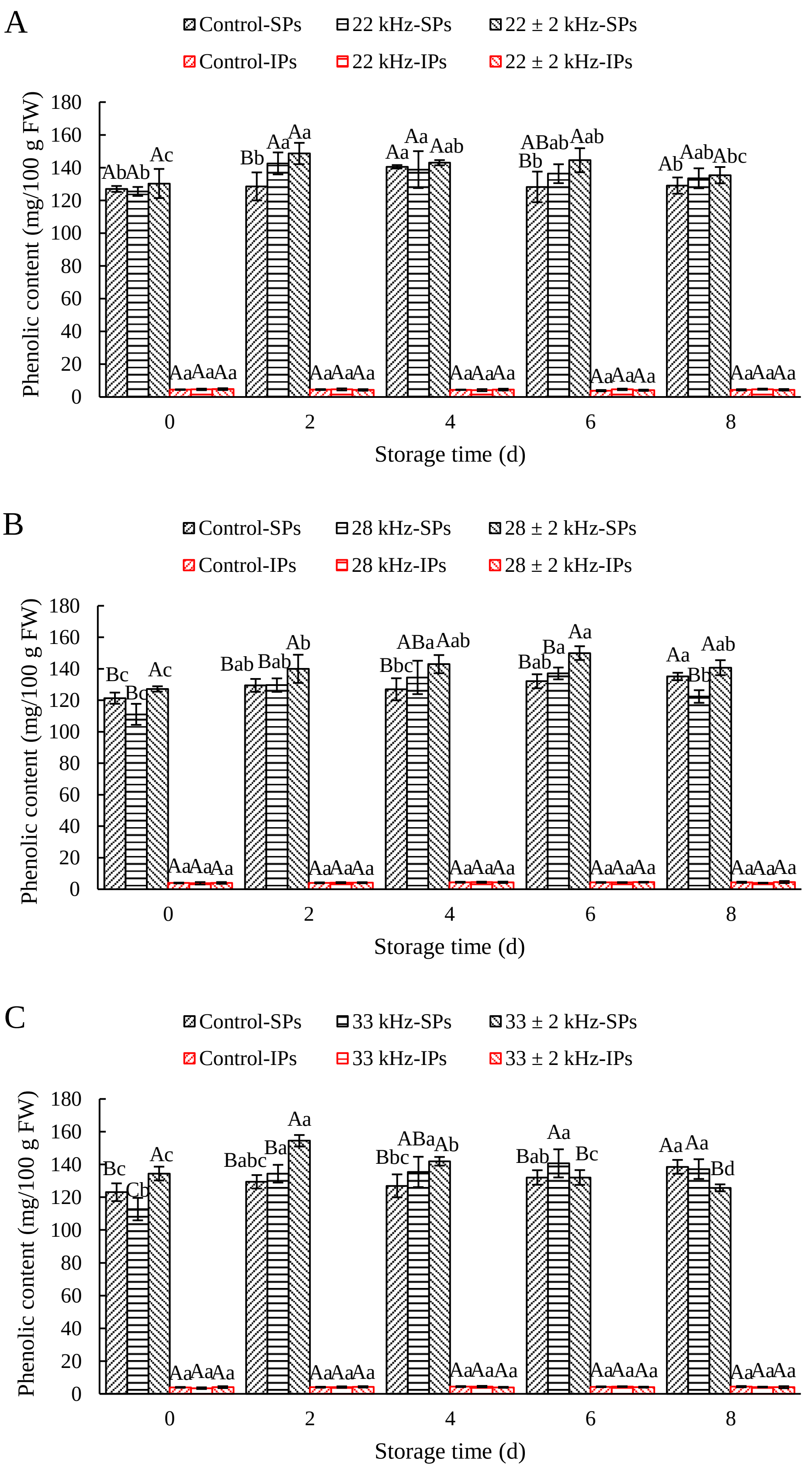
A
Control-SPs
22 kHz-SPs
22 ± 2 kHz-SPs
Control-IPs
22 kHz-IPs
22 ± 2 kHz-IPs
Phenolic content (mg/100 g FW)
0
20
40
60
80
100
120
140
160
180
AbAb Ac
Bb Aa Aa
Aa Aa Aab
Bb ABab Aab
Ab Aab Abc
AaAaAa
AaAaAa
AaAaAa
AaAaAa
AaAaAa
0
2
4
6
8
Storage time (d)
B
Control-SPs
28 kHz-SPs
28 ± 2 kHz-SPs
Control-IPs
28 kHz-IPs
28 ± 2 kHz-IPs
Phenolic content (mg/100 g FW)
0
20
40
60
80
100
120
140
160
180
Bc Bc Ac
Bab Bab Ab
Bbc ABa Aab
Bab Ba Aa
Aa Bb Aab
AaAaAa
AaAaAa
AaAaAa
AaAaAa
AaAaAa
0
2
4
6
8
Storage time (d)
C
Control-SPs
33 kHz-SPs
33 ± 2 kHz-SPs
Control-IPs
33 kHz-IPs
33 ± 2 kHz-IPs
Phenolic content (mg/100 g FW)
0
20
40
60
80
100
120
140
160
180
Bc Cb Ac
Babc Ba Aa
Bbc ABa Ab
Bab Aa Bc
Aa Aa Bd
AaAaAa
AaAaAa
AaAaAa
AaAaAa
AaAaAa
0
2
4
6
8
Storage time (d)

D

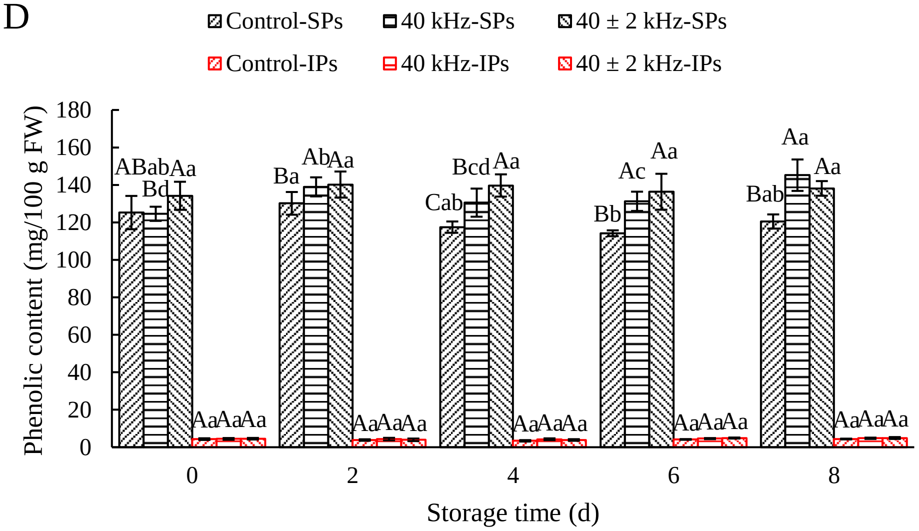

Control-SPs
40 kHz-SPs
40 ± 2 kHz-SPs
Control-IPs
40 kHz-IPs
40 ± 2 kHz-IPs
Phenolic content (mg/100 g FW)
0
20
40
60
80
100
120
140
160
180
0
2
4
6
8
Storage time (d)


E

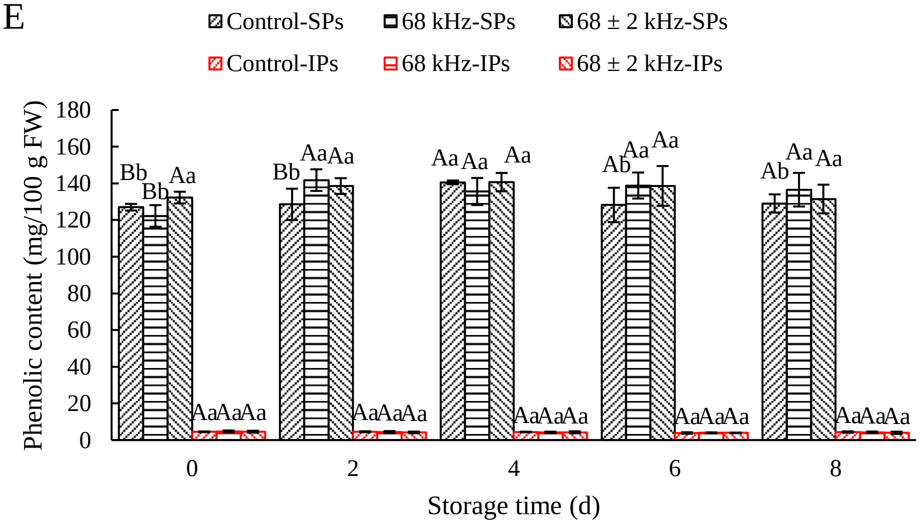

Control-SPs
68 kHz-SPs
68 ± 2 kHz-SPs
Control-IPs
68 kHz-IPs
68 ± 2 kHz-IPs
Phenolic content (mg/100 g FW)
0
20
40
60
80
100
120
140
160
180
0
2
4
6
8
Storage time (d)

**Figure S2.**

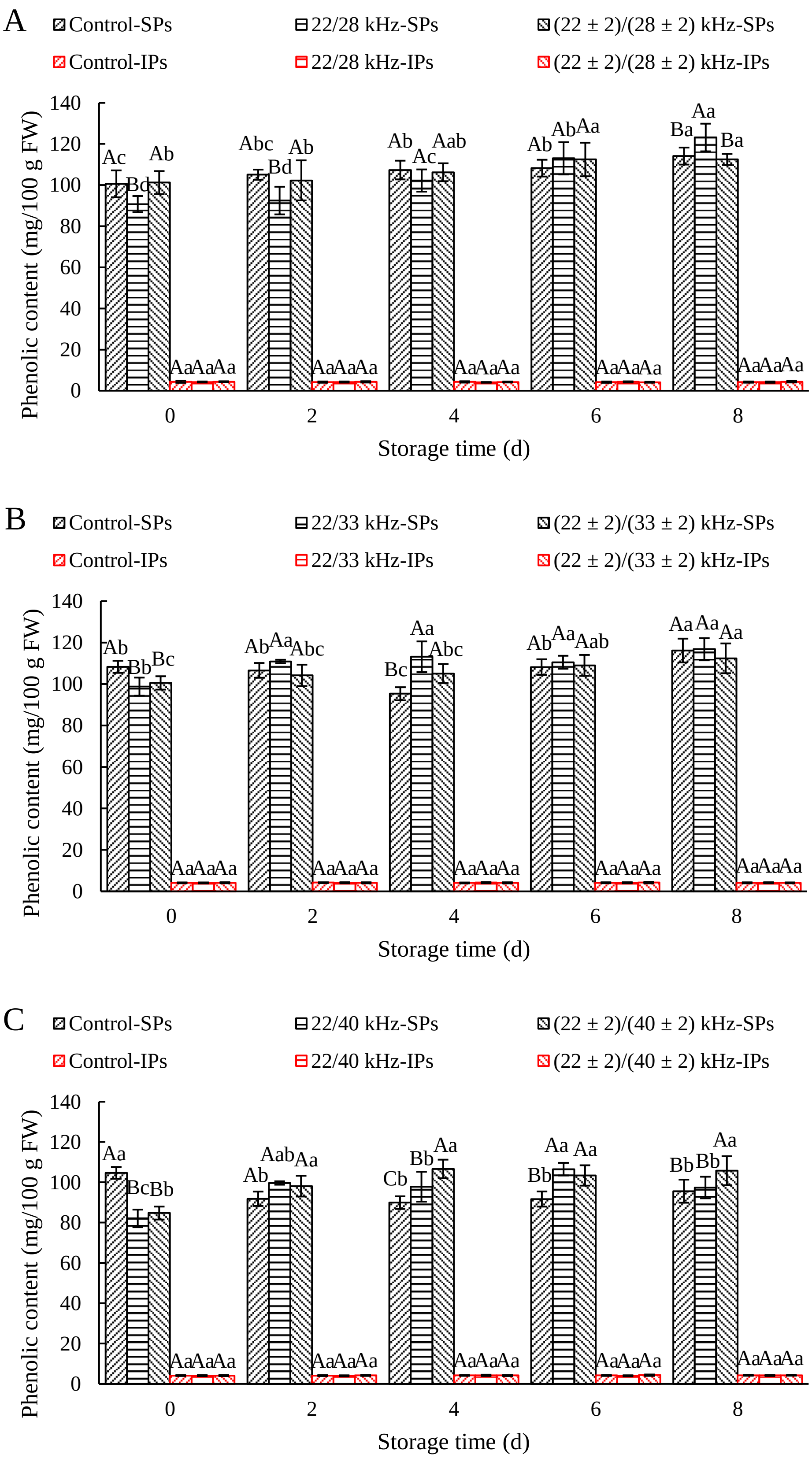
A
Control-SPs
22/28 kHz-SPs
(22 ± 2)/(28 ± 2) kHz-SPs
Control-IPs
22/28 kHz-IPs
(22 ± 2)/(28 ± 2) kHz-IPs
Phenolic content (mg/100 g FW)
Storage time (d)
B
Control-SPs
22/33 kHz-SPs
(22 ± 2)/(33 ± 2) kHz-SPs
Control-IPs
22/33 kHz-IPs
(22 ± 2)/(33 ± 2) kHz-IPs
Phenolic content (mg/100 g FW)
Storage time (d)
C
Control-SPs
22/40 kHz-SPs
(22 ± 2)/(40 ± 2) kHz-SPs
Control-IPs
22/40 kHz-IPs
(22 ± 2)/(40 ± 2) kHz-IPs
Phenolic content (mg/100 g FW)
Storage time (d)

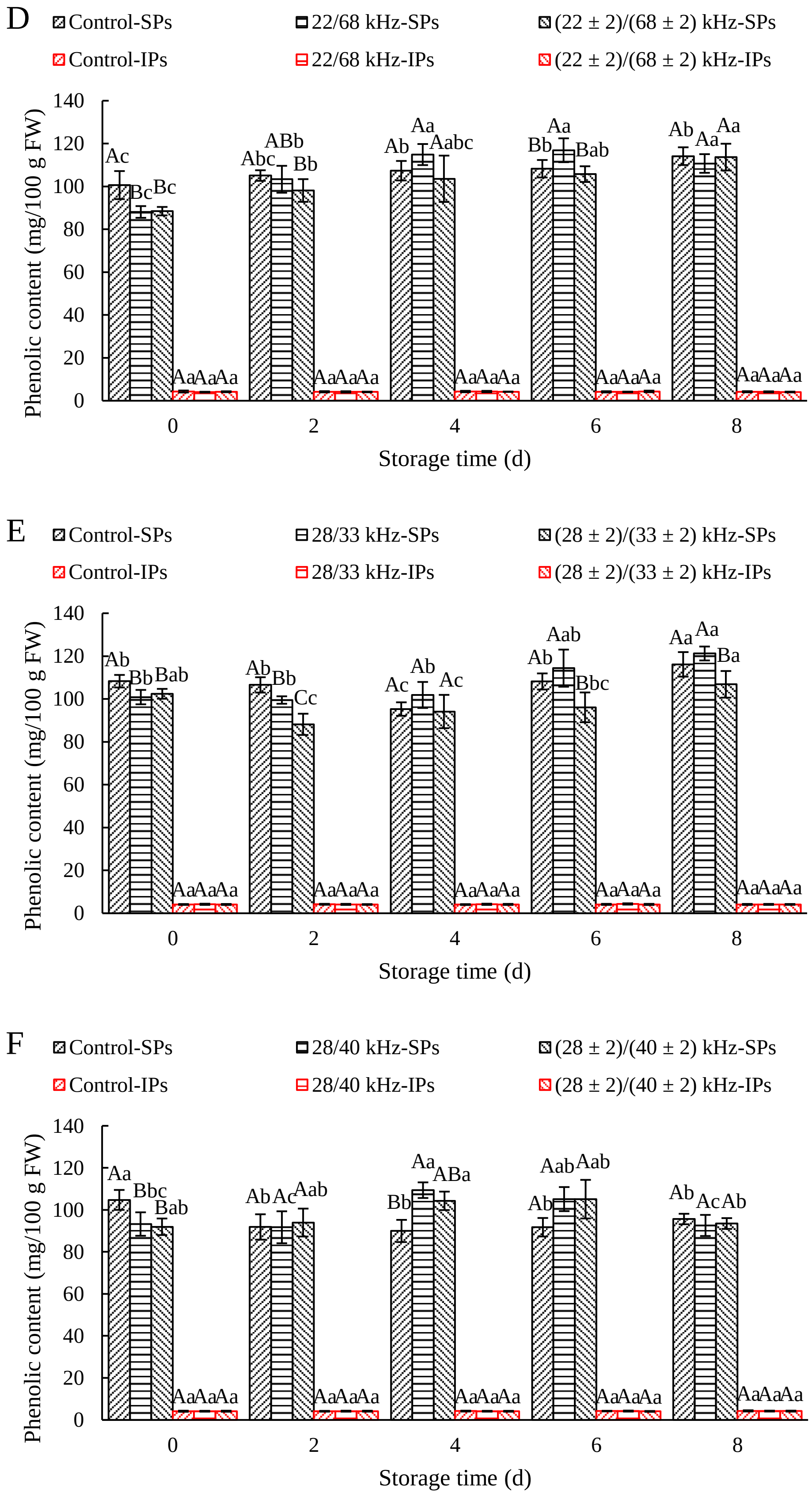

D
Control-SPs
22/68 kHz-SPs
(22 ± 2)/(68 ± 2) kHz-SPs
Control-IPs
22/68 kHz-IPs
(22 ± 2)/(68 ± 2) kHz-IPs
Phenolic content (mg/100 g FW)
Storage time (d)
E
Control-SPs
28/33 kHz-SPs
(28 ± 2)/(33 ± 2) kHz-SPs
Control-IPs
28/33 kHz-IPs
(28 ± 2)/(33 ± 2) kHz-IPs
Phenolic content (mg/100 g FW)
Storage time (d)
F
Control-SPs
28/40 kHz-SPs
(28 ± 2)/(40 ± 2) kHz-SPs
Control-IPs
28/40 kHz-IPs
(28 ± 2)/(40 ± 2) kHz-IPs
Phenolic content (mg/100 g FW)
Storage time (d)

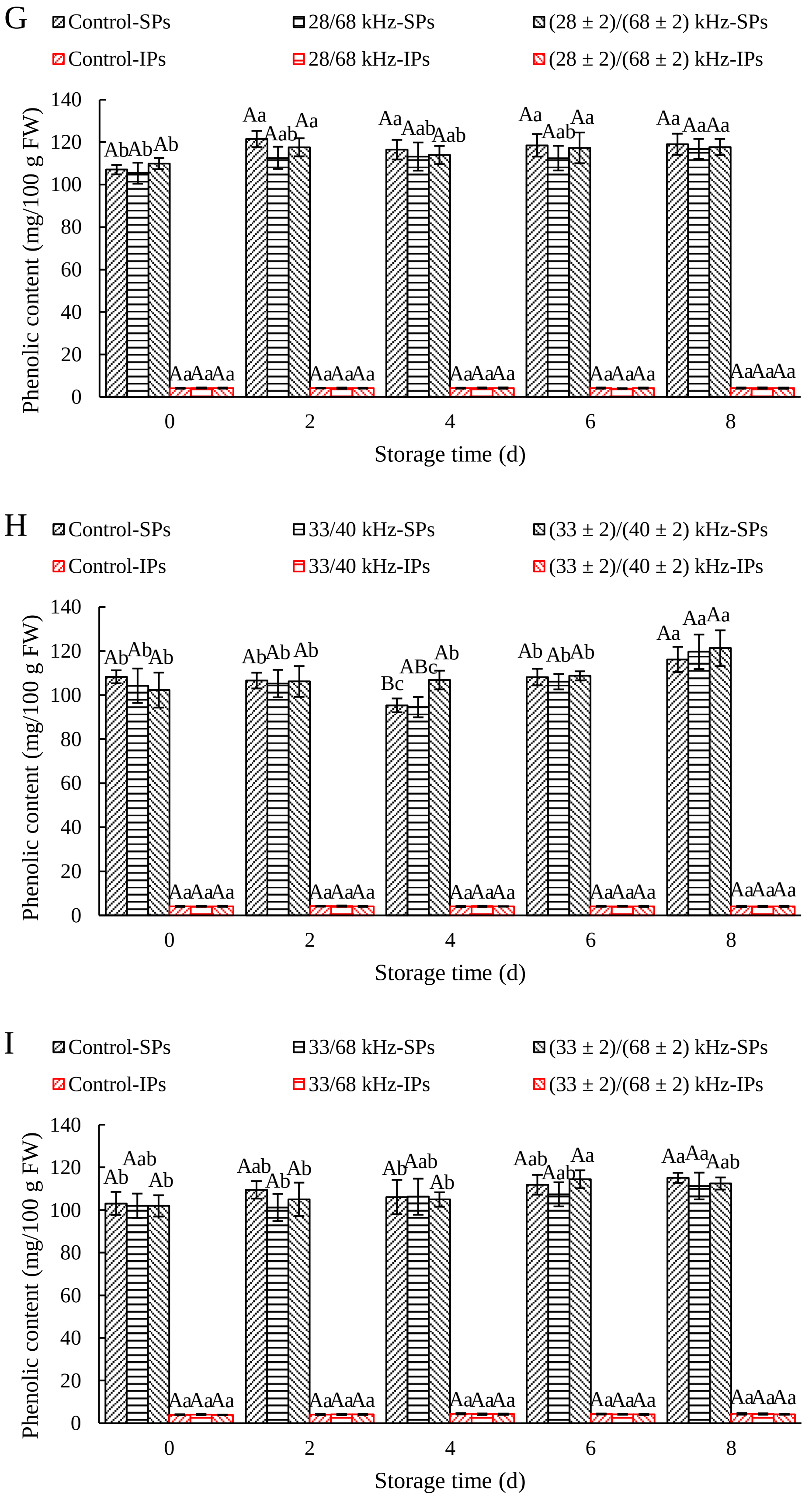
G
Control-SPs
28/68 kHz-SPs
(28 ± 2)/(68 ± 2) kHz-SPs
Control-IPs
28/68 kHz-IPs
(28 ± 2)/(68 ± 2) kHz-IPs
Phenolic content (mg/100 g FW)
Storage time (d)
H
Control-SPs
33/40 kHz-SPs
(33 ± 2)/(40 ± 2) kHz-SPs
Control-IPs
33/40 kHz-IPs
(33 ± 2)/(40 ± 2) kHz-IPs
Phenolic content (mg/100 g FW)
Storage time (d)
I
Control-SPs
33/68 kHz-SPs
(33 ± 2)/(68 ± 2) kHz-SPs
Control-IPs
33/68 kHz-IPs
(33 ± 2)/(68 ± 2) kHz-IPs
Phenolic content (mg/100 g FW)
Storage time (d)

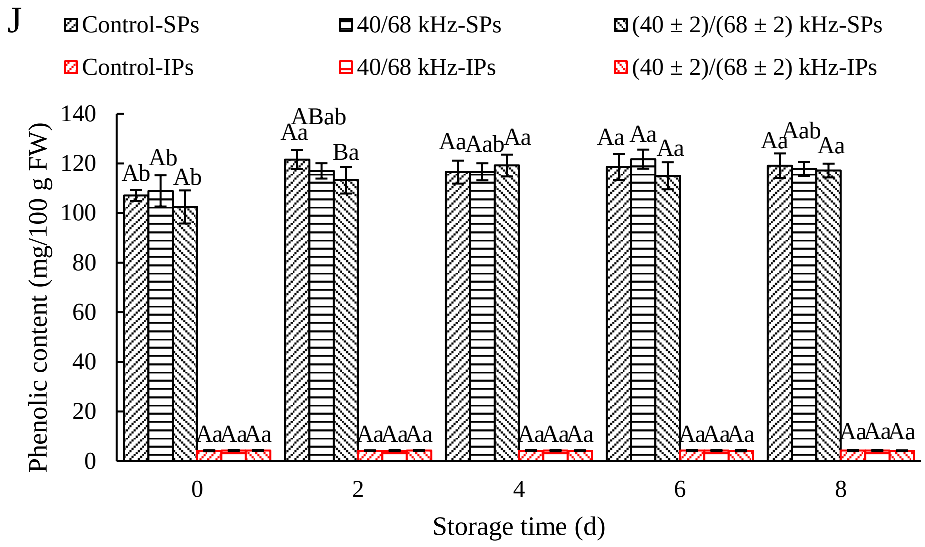
J
Control-SPs
40/68 kHz-SPs
(40 ± 2)/(68 ± 2) kHz-SPs
Control-IPs
40/68 kHz-IPs
(40 ± 2)/(68 ± 2) kHz-IPs
Phenolic content (mg/100 g FW)
Storage time (d)
0
2
4
6
8
20
40
60
80
100
120
140
Ab
Ab
Ab
Aa
ABab
Ba
Aa
Aab
Aa
Aa
Aa
Aa
Aa
Aab
Aa
AaAaAa

**Figure S3.**

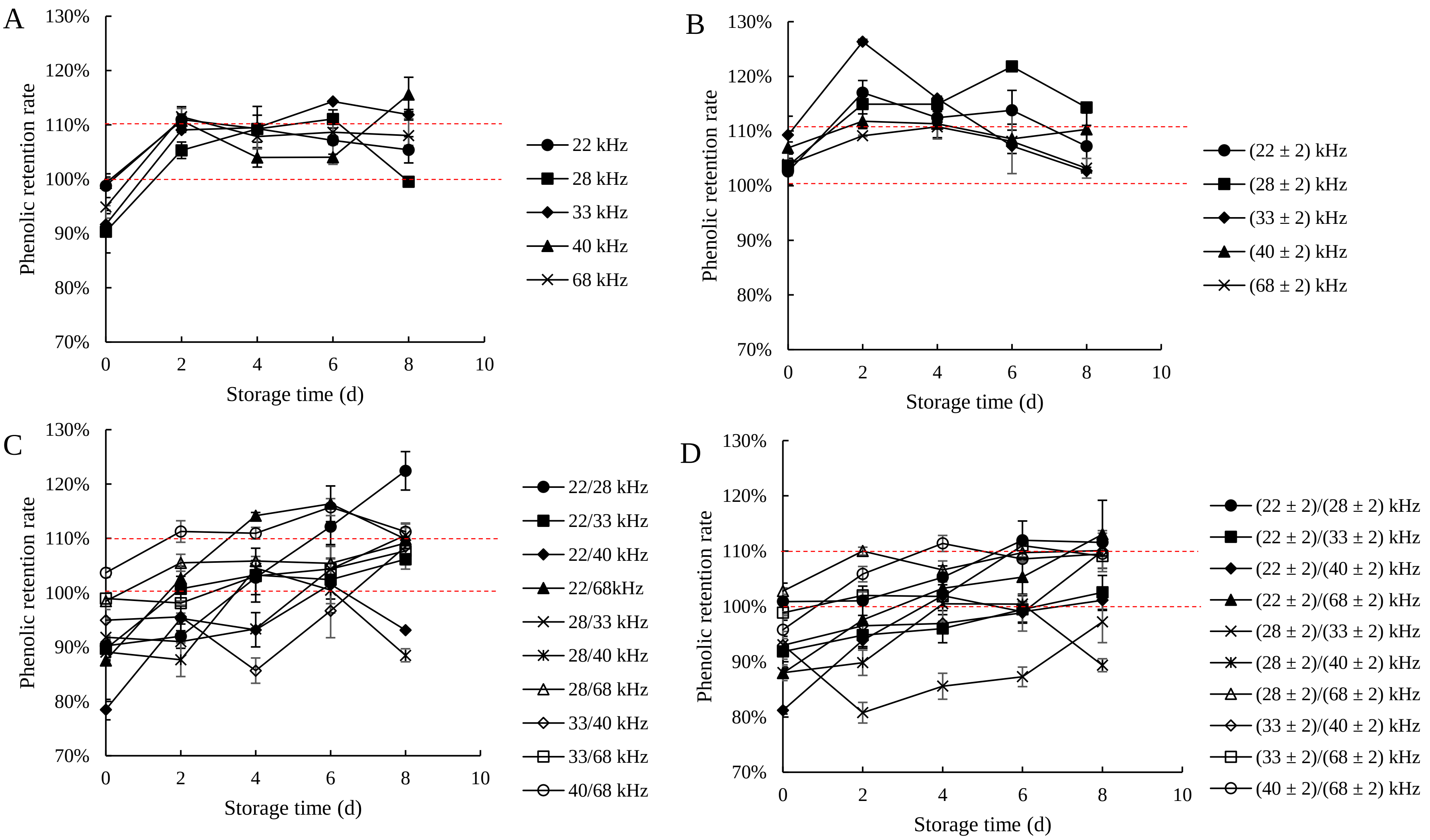

A
B
C
D
Phenolic retention rate
Storage time (d)
130%
120%
110%
100%
90%
80%
70%
0
2
4
6
8
10
22 kHz
28 kHz
33 kHz
40 kHz
68 kHz
(22 ± 2) kHz
(28 ± 2) kHz
(33 ± 2) kHz
(40 ± 2) kHz
(68 ± 2) kHz
22/28 kHz
22/33 kHz
22/40 kHz
22/68kHz
28/33 kHz
28/40 kHz
28/68 kHz
33/40 kHz
33/68 kHz
40/68 kHz
(22 ± 2)/(28 ± 2) kHz
(22 ± 2)/(33 ± 2) kHz
(22 ± 2)/(40 ± 2) kHz
(22 ± 2)/(68 ± 2) kHz
(28 ± 2)/(33 ± 2) kHz
(28 ± 2)/(40 ± 2) kHz
(28 ± 2)/(68 ± 2) kHz
(33 ± 2)/(40 ± 2) kHz
(33 ± 2)/(68 ± 2) kHz
(40 ± 2)/(68 ± 2) kHz

**Figure S4.**

A B

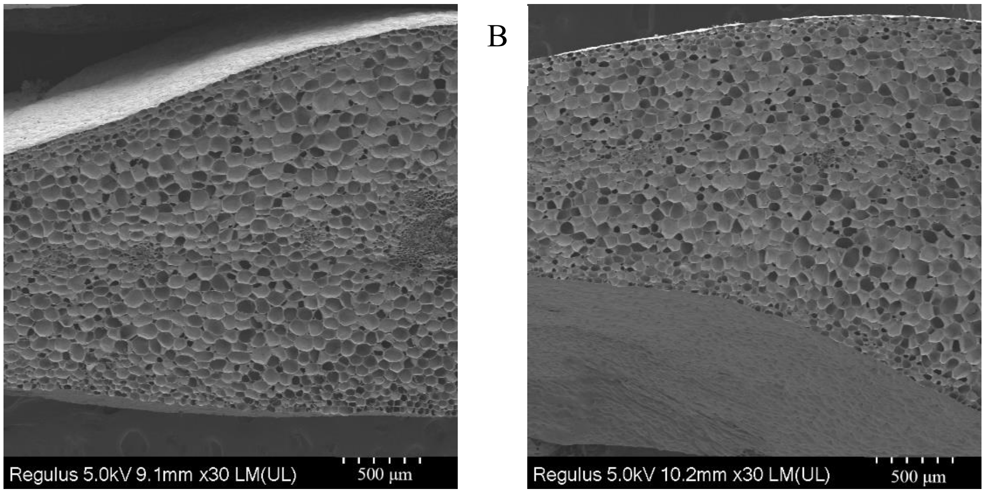
Regulus 5.0kV 9.1mm x30 LM(UL)
500 µm
Regulus 5.0kV 10.2mm x30 LM(UL)
500 µm

**Figure S5.**

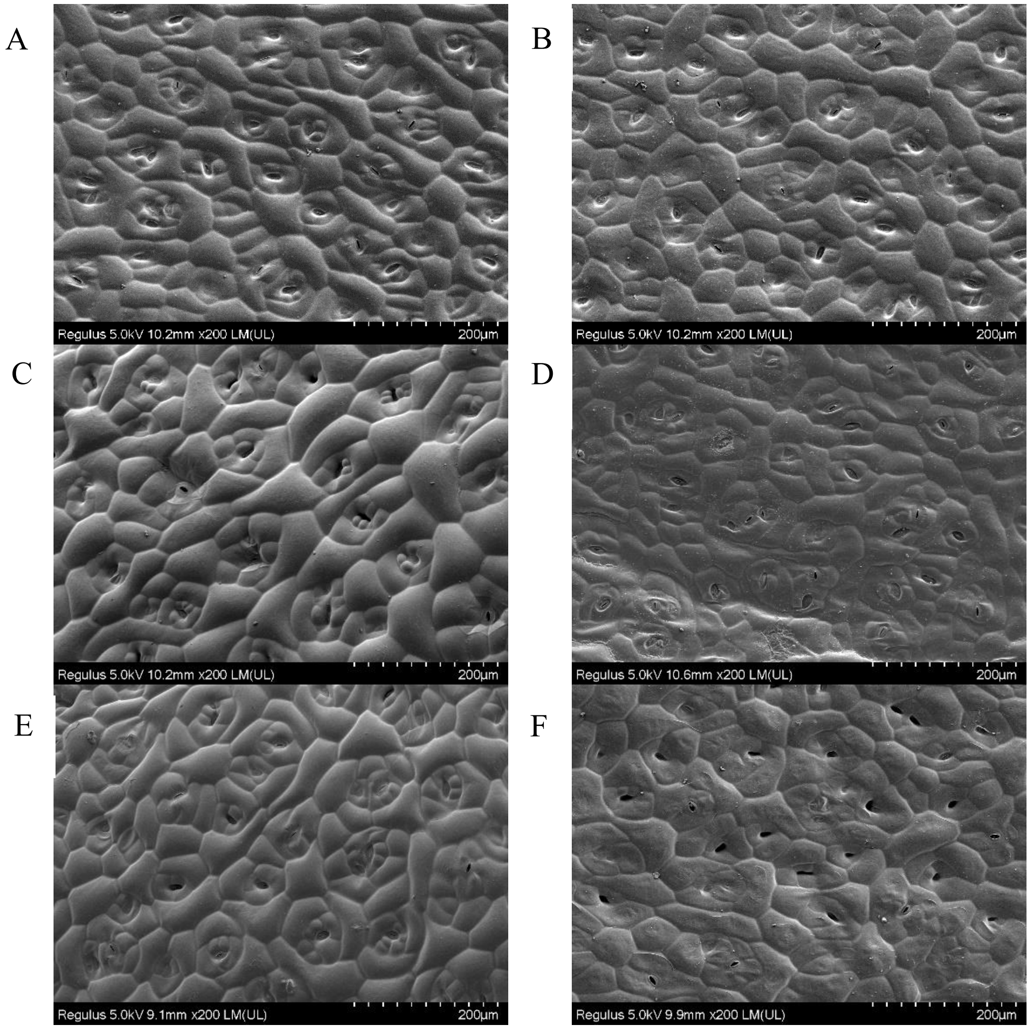
A
B
Regulus 5.0kV 10.2mm x200 LM(UL)
200µm
Regulus 5.0kV 10.2mm x200 LM(UL)
200µm
C
D
Regulus 5.0kV 10.2mm x200 LM(UL)
200µm
Regulus 5.0kV 10.6mm x200 LM(UL)
200µm
E
F
Regulus 5.0kV 9.1mm x200 LM(UL)
200µm
Regulus 5.0kV 9.9mm x200 LM(UL)
200µm

**Figure S6.**

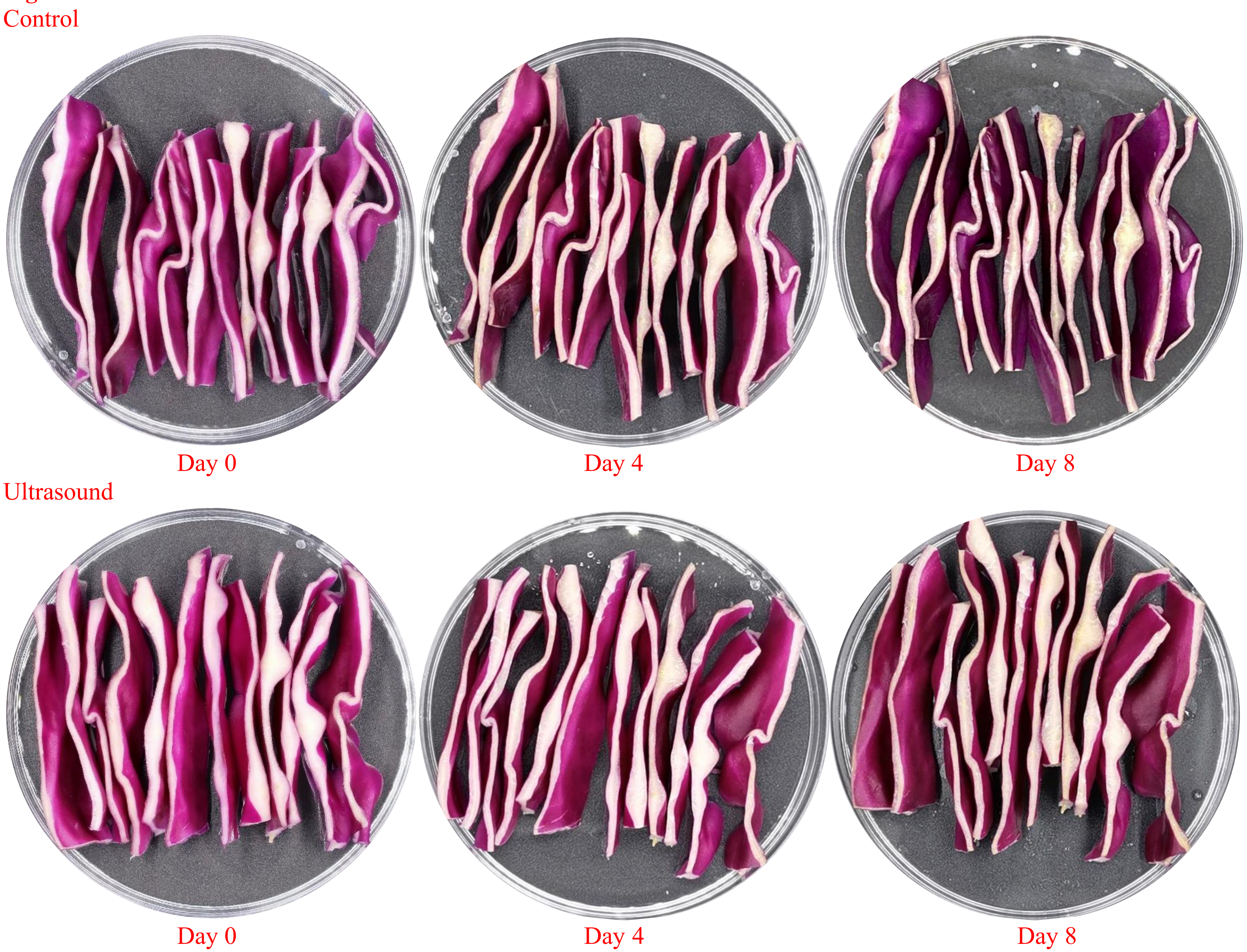

Supplement: Supplementary file 1 [file Data_Sheet_1.pdf]
